# Interphase human chromosome exhibits out of equilibrium glassy dynamics

**DOI:** 10.1038/s41467-018-05606-6

**Published:** 2018-08-08

**Authors:** Guang Shi, Lei Liu, Changbong Hyeon, D. Thirumalai

**Affiliations:** 1 Biophysics Program, Institute for Physical Science and Technology, University of Maryland, College Park, MD 20742 USA; 2 Korea Institute for Advanced Study, Seoul, 02455 Republic of Korea; 3 Department of Chemistry, University of Texas at Austin, Austin, TX 78712 USA

## Abstract

Fingerprints of the three-dimensional organization of genomes have emerged using advances in Hi-C and imaging techniques. However, genome dynamics is poorly understood. Here, we create the chromosome copolymer model (CCM) by representing chromosomes as a copolymer with two epigenetic loci types corresponding to euchromatin and heterochromatin. Using novel clustering techniques, we establish quantitatively that the simulated contact maps and topologically associating domains (TADs) for chromosomes 5 and 10 and those inferred from Hi-C experiments are in good agreement. Chromatin exhibits glassy dynamics with coherent motion on micron scale. The broad distribution of the diffusion exponents of the individual loci, which quantitatively agrees with experiments, is suggestive of highly heterogeneous dynamics. This is reflected in the cell-to-cell variations in the contact maps. Chromosome organization is hierarchical, involving the formation of chromosome droplets (CDs) on genomic scale, coinciding with the TAD size, followed by coalescence of the CDs, reminiscent of Ostwald ripening.

## Abstract (web summary)

The 3D organization of chromosomes within the nuclear space is important for biological functions. Here the authors model chromosomes as self-avoiding copolymers with distinct epigenetic states, and show that the features of experimentally generated contact maps can be reproduced and their dynamics predicted.

## Introduction

The organization of chromosomes without topological entanglement or knot formation in the crowded tight space of the nucleus is remarkable. Understanding the structural organization and the dynamics of eukaryotic chromosomes and the mechanism of chromosome territories formation may hold the key to enunciating genome functions^1,2^. Glimpses into the structures of the chromosomes have emerged, thanks to spectacular advances in chromosome conformation capture (3C, 4C, 5C, and Hi-C) experiments^3–5^, from which the probability, *P*_*ij*_, that any two loci *i* and *j* are in contact can be inferred. The set of *P*_*ij*_, which is a two-dimensional representation of the spatial organization, constitutes the contact map. More recently, imaging methods like FISH^6^ as well as super-resolution technique^7,8^ have more directly determined the positions of the loci of single chromosomes, thus providing a much-needed link to the indirect measure of spatial organization revealed through contact maps. The experiments by Zhuang and coworkers and others^6–8^ are of great value because the number of constraints needed to unambiguously infer structures from contact maps alone is very large^9^.

Contact maps, constructed from Hi-C experiments, revealed that chromosome is organized into compartments on genomic length scales exceeding megabases (Mbps)^4,5^. The partitioning of the structure into compartments are highly correlated with histone markers of the chromatin loci^5^, implying that contacts are enriched within a compartment and depleted between different compartments. The loci associated with active histone markers and those associated with repressive histone markers localize spatially in different compartments. Higher resolution Hi-C experiments^5^ have also identified topologically associated domains (TADs) on scales on the order of hundreds of kilobases^3^. The TADs are the square patterns along the diagonal of the contact maps in which the probability of two loci being in contact is more probable than between two loci belonging to distinct TADs. Multiplexed FISH experiments^6^ show most directly that TADs belonging to distinct compartments are spatially separated in the chromosomes.

The experimental studies have inspired a variety of polymer models^10–25^, which have provided insights into many aspects of chromosome organization. These studies are particularly important because finding a unique solution (if one exists) to the inverse problem of deriving spatial structures from contact maps is most challenging^9^. Some of the features in the contact maps, such as the probability *P*(*s*) that two loci separated by a certain genomic distance (*s*) are in contact, may be computed using a homopolymer model^4^, without accounting for the epigenetic states, whereas fine structures such as TADs and compartments require copolymer or heteropolymer models^18,20,21,26^.

Biological functions, such as the search for genes by transcription factors or mechanism for DNA damage repair, not only depend on genome structure but also the associated dynamics. The use of polymer models in describing chromatin structure has a rich history^10,11^. More recent studies show that polymer physics concepts have been most useful in predicting the probabilistic nature of chromosome organization inferred from Hi-C experiments^12–23^. In contrast, the dynamic aspects of the interphase chromosome have received much less attention^24–29^. Experiments have revealed that genome-wide chromatin dynamics^29–32^ of chromatin fiber in mammalian cells exhibit heterogeneous sub-diffusive behavior. Thus, it is important to understand how the slow dynamics of the individual locus and long length scale coherent collective motions emerge from the highly organized chromosomes.

Here, we develop a copolymer model to describe both the structure and dynamics of human interphase chromosomes based on the assumption that the large-scale organization of human interphase chromosome is largely driven and maintained by the interactions between the loci of similar epigenetic states. Similar models, that differ greatly in details, have been developed to model the 3D structure of Drosophila chromosomes^18,20^. Jost et al.^18^ used a heteropolymer model with four different types of monomers representing active, Polycomb, HP1 and black chromatin to describe the formation of TADs in *Drosophila* genome. Michieletto et al.^26^ constructed a heteropolymer with three epigenetic states (acetylated, methylated, and unmarked) to probe how the epigenetic states are maintained. A very different reverse-engineering approach, with Hi-C contact maps as inputs, was used to construct an energy function with 27 parameters^21^. We take a “bottom-up” approach to incorporate the epigenetic states into the polymer model similar in spirit to the previous studies^18,20,26^. We show that in order to capture the structural features faithfully, at least two types of beads, representing active and repressive loci are needed. Simulations of the resulting chromosome copolymer model (CCM) for human interphase chromosomes 5 and 10 show that the structural characteristics, such as the scaling of *P*(*s*) as a function of *s*, compartments, and TADs indicated in the Hi-C contact maps are faithfully reproduced. We use sophisticated clustering algorithms to quantitatively compare the simulated contact maps and those inferred from Hi-C experiments. The compartment feature noted in the Hi-C contact map is due to micro-phase separation between chromosome loci associated with different epigenetic states, implying that a copolymer model is needed for characterizing large-scale genome organization. The TADs emerge by incorporating experimentally inferred positions of the loop anchors, whose formation is facilitated by CTCF motifs. The only free parameter in the CCM, the optimal loci–loci interaction strength between loci belonging to the same epigenetic states, is adjusted to give a good description of the Hi-C contact map. Using simulations based on the resulting CCM, we show that chromosome dynamics is highly heterogeneous and exhibits many of the characteristics of out of equilibrium glassy dynamics, with coherent motion on μm scale, including stretched exponential decay of the scattering function (*F*_s_(*k*,*t*)), a non-monotonicity behavior in the time dependence of the fourth order susceptivity associated with fluctuations in *F*_s_(*k*,*t*). Of particular note is the remarkable cell-to-cell and loci-to-loci variation in the time (*t*) dependence of the mean square displacement, Δ_*i*_(*t*), of the individual loci. The distribution *P*(*α*) of the exponent associated with the increase in Δ_*i*_(*t*) ~ *t*^*α*^ is broad. The simulated and experimentally measured *P*(*α*)s are in excellent agreement. Our work shows that chromosomes structures are highly dynamic exhibiting large cell-to-cell variations in the contact maps and dynamics. The rugged chromosome energy landscape, with multiple minima separated by large barriers, is perhaps needed to achieve a balance between genomic conformational stability and dynamics for the execution of a variety of biological functions.

## Results

### Choosing the energy scale in the CCM

**Fig. 1:**
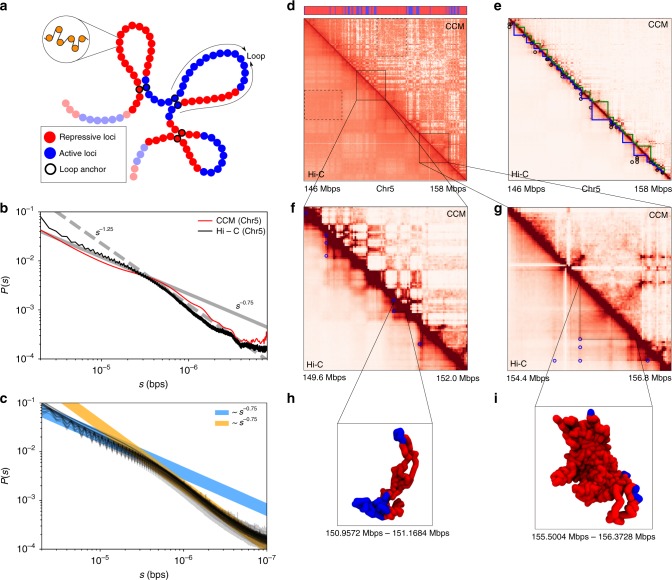
Comparison between the simulated contact map and the Hi-C contact map. **a** A sketch of the chromosome copolymer model (CCM). Each bead represents 1200 base pairs (representing roughly six nucleosomes connected by linker DNAs). Blue (red) corresponds to active (repressive) loci. The examples of three pairs of loop anchors (in this cartoon) are marked by beads with black boundaries. **b** Comparison between experimental data^5^ (black) and simulated *P*(*s*). Dashed and solid lines are plots of *s*^−1.25^ and *s*^−0.75^, respectively. The crossover point between the two scaling regimes at s* ~ 3 × 10^5^ bps is noticeable in both the experimental and simulated results. **c** Experimental contact probability *P*(*s*) for the 23 human interphase chromosomes calculated from the Hi-C data in ref. ^5^ Each black curve, all of which almost superimpose on each other, corresponds to one chromosome. Blue and orange lines are guides to the eye showing two scaling regimes. **d** Comparison of the contact maps inferred from Hi-C experiment^5^ (lower triangle) and obtained from simulations (upper triangle) results. For easier visualization, the values of the contact probability are converted to a log_2_ scale. The bar above the map marks the epigenetic states with blue (red) representing active (repressive) loci. The dashed black box is an example of a compartment. Such compartment-like structures emerge due to contacts between loci separated by large genomic distances, which gives rise to spatial order in the organized chromosome. **e** Illustration of topologically associated domains (TADs). The blue and green triangles are from experiments and simulations, respectively. The black circles mark the positions of loops detected from experiment data, which are formed by two CTCF motifs. **f** The zoom in of the diagonal region for the chromosome segment between 149.6 and 152.0 Mbps. The blue circle marks the positions of CTCF loops found in the experiment^5^. **g** Same as **f** except for 154.4–156.8 Mbps. **h**, **i** Snapshots of two TADs, marked by the  black triangles in **f** and **g**, respectively

We fixed *N*, the size of the copolymer to *N* = 10,000, modeling a 12 Mbps (megabases) chromatin fiber, corresponding to a selected region of the Human Cell line GM12878 Chromosome 5 (Chr 5) from 145.87 Mbps to 157.87 Mbps. In the CCM (Fig. 1a and Supplementary Fig. 1), the only unknown parameter is $\epsilon$, characterizing the strength of the interaction between the loci (Supplementary Table I). We chose a $\epsilon$ value that reproduces the contact maps that is near quantitative agreement with the Hi-C data. As $\epsilon$ increases, the structures of the chromosome are arranged in such a way that segments with small genomic distance *s* are more likely to be in spatial proximity (see the section “Chromosome Structures in terms of WLM” below). This is also illustrated in Supplementary Fig. 4, which shows that higher values of $\epsilon$ lead to clearer segregation between the loci with different colors. The colors encode the genomic locations. The snapshots of the organized chromosome, the good agreement between the calculated and Hi-C contact maps, and the accurate description of the spatial organization as assessed by the ward linkage matrix (WLM) (Supplementary Note 9) confirm that $\epsilon$ = 2.4*k*_B_*T* produces the closest agreement with experiments. Increasing $\epsilon$ beyond 2.4*k*_B_*T* leads to a worse description of segregation between loci with distinct epigenetic states.

Furthermore, *P*(*s*) as a function of *s* obtained in simulations with $\epsilon$ = 2.4*k*_B_*T* is also consistent with experiments (see below). The *s*-dependent contact probability, *P*(*s*) in Fig. 1b, shows that there are two scaling regimes. As $\epsilon$ increases, the probability of short-range (small *s*) increases by several folds, while *P*(*s*) for large *s* decreases by approximately an order of magnitude (Supplementary Fig. 13a). In particular, for $\epsilon$ = 1.0*k*_B_*T*, *P*(*s*), decreases much faster compared to experiments at small *s*. In contrast, we find that at $\epsilon$ = 2.4*k*_B_*T*, *P*(*s*) ~ *s*^−0.75^ for *s* < 0.5 Mbps and when *s* exceeds ~0.5 Mbps, *P*(*s*) ~ *s*^−1.25^ (red curve in Fig. 1b). Such a behavior, with *P*(*s*) exhibiting two distinct scaling regimes, agrees with experiments (black line in Fig. 1b). It is worth pointing out that the two scaling regimes in *P*(*s*) is a robust feature of all 23 human interphase chromosomes (Fig. 1c). It is clear the two scaling regimes in *P*(*s*) with a crossover from one to another at *s* ≈ 3 × 10^5^–6 × 10^5^ bps is universally found in all the chromosomes. Interestingly, our simulation suggests that the crossover scale in *P*(*s*) coincides with the size of the chromosome droplets (see discussion).

### Active and repressive loci micro-phase segregate

Comparison of the contact maps between simulations and experiments illustrates that compartment formation appearing as plaid or checkerboard patterns in Fig. 1d, shows good agreement with Hi-C data^4,5^. The dashed rectangles mark the border of one such compartment enriched predominantly with interactions between loci of the same type, suggesting that compartments are formed through the clustering of the chromatin segments with the same epigenetic states. A previous experimental study suggests that the chromatin structuring in TADs is also driven by the epigenome feature^33^. In order to make the comparison precise, we treated the contact maps as probabilistic matrices and used a variety of mathematical methods to quantitatively compare large matrices. First, the checkerboard pattern in the contact map is more prominent when illustrated using the Spearman correlation map (see Supplementary Note 7 and Supplementary Figs. 6 and 7). Second, to quantitatively compare the simulated results with experiments, we use the spectral co-clustering algorithm^34^ to bi-cluster the computed Spearman correlation map (see Supplementary Note 8). Other methods, such as PCA^4^ and *k*-means^5^, have been used to extract the compartment features. The spectral co-clustering deployed here, gives results that closely resemble those obtained using PCA (Supplementary Fig. 20). Finally, the similarity between the simulated and experimental data is assessed using the Adjusted Mutual Information Score (AMI) (Supplementary Note 8). The CCM model, based only on epigenetic information and the locations of the loop anchors, yields an AMI score that results in correctly reproducing ≈81% of the compartments obtained from the experimental data. In contrast, a pseudo homopolymer model with $\epsilon _{{\mathrm{AA}}} = \epsilon _{{\mathrm{BB}}} = \epsilon _{{\mathrm{AB}}} = \epsilon$, which has the same loop anchors as the CCM, has an absolute AMI score that is 200 times smaller (Supplementary Fig. 9), and does not lead to the formation of compartments (correctly reproducing only ≈51% of the compartments, no better than random assignments). Thus, the CCM is the minimal model needed to reproduce the essential features found in the contact map.

**Fig. 2:**
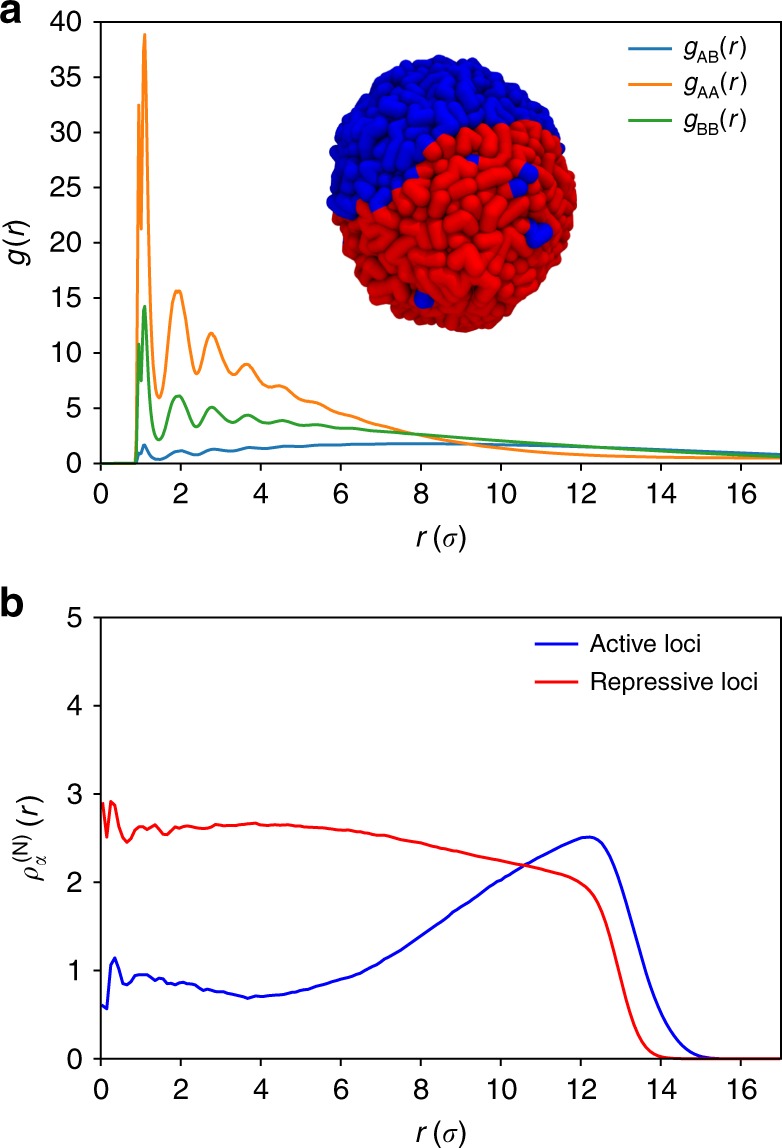
Micro-phase separation between active and repressive loci. **a** Radial distribution functions, *g*(*r*), as a function of *r* (in the unit of *σ*) between active–active loci (*g*_AA_(*r*)), repressive–repressive loci (*g*_BB_(*r*)) and active–repressive loci (*g*_AB_(*r*)). The inset shows the typical conformation of the compact chromosome. Blue and red segments correspond to active and repressive loci, respectively. The structure vividly reveals micro-phase separation between active and repressive loci. **b** The normalized radial density, $\rho _\alpha ^{({\mathrm{N}})}(r) = \langle N_\alpha (r)\rangle V/(4\pi r^2\Delta rN_\alpha )$, where *N*_*α*_(*r*) is the number of loci of given type *α* found in the spherical shell between *r* and *r* + Δ*r*, *N*_*α*_ is the total number of loci of that type. The bracket $\langle \cdot \rangle$ is the ensemble average, *V* is the volume of the globule, given by $(4/3)\pi r_{{\mathrm{max}}}^3$, where *r*_max_ = 17*σ*; $\rho _\alpha ^{({\mathrm{N}})}(r)$ shows that the active loci are predominantly localized on the periphery of the condensed chromosome. The repressive loci are more uniformly distributed

The inset in Fig. 2a, displaying a typical snapshot of the condensed chromosome, reveals that active (A, blue) and repressive (B, red) loci are clustered together, undergoing micro-phase separation (see Methods for definition of active and repressive loci). The tendency to segregate is vividly illustrated in the radial distribution functions *g*_AA_(*r*), *g*_BB_(*r*), and *g*_AB_(*r*), which shows (Fig. 2a) that *g*_AA_(*r*) and *g*_BB_(*r*) have much higher values than *g*_AB_(*r*) implying that active and repressive loci form the clusters of their own, and do not mix. Such a micro-phase separation between the A-rich and B-rich regions directly gives rise to compartments in the contact map. Interestingly, the normalized radial density (Fig. 2b) shows that active chromatin exhibits a peak at large radial distance, *r,* implying that the active loci localize on the periphery of the condensed chromosome whereas repressive chromatin is more homogeneously distributed. Visual inspection of the simulation trajectories also suggests that active and repressive chromatins are often separated in a polarized fashion, in accord with a recent experimental study^6^, which shows that the two compartments are indeed similarly spatially arranged.

### Spatial organization of the compact chromosome

**Fig. 3:**
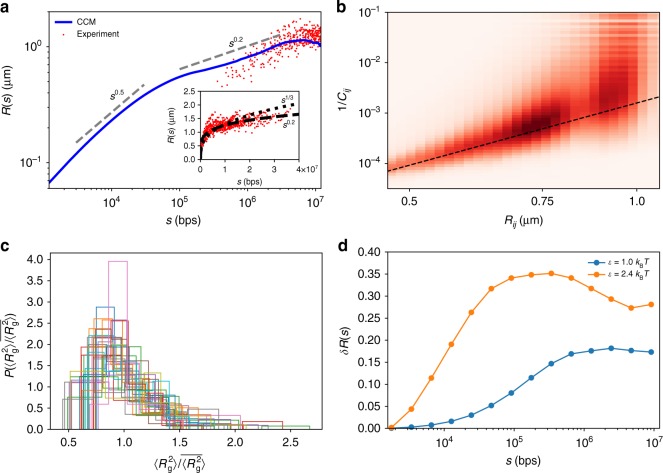
Organization and fluctuations of the chromosome structures. **a** The dependence of the spatial distance *R*(*s*) (Eq.1) on the genomic distance, *s*. Gray dashed lines, indicating the slopes, are guides to the eye. The red dots are experimental data taken from^6^ for *s* < 1.2×10^7^bps. The inset shows the complete set of experimental data. Short dashed and long dashed lines are *s*^1/3^ and *s*^0.2^, respectively. At small *s* (*s* < 10^5^bps), *R*(*s*) ~ *s*^0.5^ implying that chromatin behaves as almost an ideal chain. **b** The heatmap of the 2D histogram of (*R*_*ij*_,1/*C*_*ij*_). The dashed black line is the curve with scaling exponent 4.1, which coincides with the value obtained by fitting the experimental data^6^. **c** Distribution $P(\langle R_{\mathrm{g}}^2\rangle /\overline {\langle R_{\mathrm{g}}^2\rangle } )$, where $\langle R_{\mathrm{g}}^2\rangle$ is the time average value of the squared radius of gyration of a single trajectory and $\overline {\langle R_{\mathrm{g}}^2\rangle }$ is the mean value averaged over all independent trajectories. Different colors represent $P(\langle R_{\mathrm{g}}^2\rangle /\overline {\langle R_{\mathrm{g}}^2\rangle } )$ for the 32 individual TADs. The distribution is surprisingly wide, which suggests that TAD structures vary from cell-to-cell. **d** Coefficient of variation *δR*(*s*) = $(\langle R^2(s)\rangle - \langle R(s)\rangle ^2)^{1/2}/\langle R(s)\rangle$, computed from simulations, shows a non-monotonic dependence on *s* for $\epsilon$ = 2.4*k*_B_*T*, increasing till *s* ~ 10^5^ bps and decreases at larger *s*

In order to illustrate the spatial organization of the chromosome, we introduce the distance function,

1 $$R(s) = \left\langle {\mathop {\sum}\limits_{i < j}^N \frac{{({\mathbf{r}}_i - {\mathbf{r}}_j)^2\delta (s - |i - j|)}}{{N - s}}} \right\rangle ^{1/2}$$

where $\langle \cdot \rangle$ denotes both an ensemble and time average. We calculated *R*(*s*), the mean end-to-end distance between the loci, by constraining the genomic distance $|i - j|_{}^{}$ to *s*. If the structured chromosome is maximally compact on all length scales, we expect *R*(*s*) ~ *s*^1/3^ for all *s*. However, the plot of *R*(*s*) on a log-log scale shows that in the range 10^5^ ≲ *s* ≲ 10^6^ bps, *R*(*s*) ~ *s*^0.2^. The plateau at large *s* arises due to *s* reaching the boundary of the compact structure. The inset in Fig. 3a, comparing the simulation result and experimental data^6^, both show the same scaling for *R*(*s*) as a function of *s*. Note that in ref. ^6^ spatial distances are measured between centroids of TADs domains rather than individual loci. We present the equivalence between Eq. (1) and the distances between the TAD centroids in Supplementary Note 6.

By a systematic analysis of the FISH data, Wang et al.^6^ established that the probability of contact formation between loci *i* and *j*, *P*_*ij*_, is inversely proportional to a power of their mean spatial distance *R*_*ij*_ = $\langle |{\mathbf{r}}_i - {\mathbf{r}}_j|\rangle$, with the latter providing a direct picture of the spatial organization. Similarly, in this work, we explored the relation between *C*_*ij*_ and *R*_*ij*_ where $C_{ij}\left( { = P_{ij}\mathop {\sum}\nolimits_{i < j} C_{ij} \propto P_{ij}} \right)$ is the number of contacts between loci *i* and *j* that are recorded in the simulations. The heatmap of (1/*C*_*ij*_,*R*_*ij*_) in Fig. 3b shows that the two matrices are proportional to each other. In accord with the FISH data^6^, we find that $1/C_{ij} \propto R_{ij}^\lambda$ where *λ* ≈ 4, suggesting that larger mean spatial distance between loci *i* and *j* implies smaller contact probability, which is the usual assumption when experimental Hi-C data are used to infer three-dimensional chromosome organization. The decrease of *C*_*ij*_ with increasing *R*_*ij*_ with a large value of *λ*, is unexpected but is an important finding needed to link contact maps and spatial structures.

The slope of the dashed line in Fig. 3b obtained using the data in ref. ^6^, is 4.1, which coincides with our simulation results. Mean-field arguments^35^ suggest that *P*(*s*) ~ *R*(*s*)^−3^, which follows from the observation that the end of the chain is uniformly distributed over a volume *R*^3^(*s*). This is neither consistent with our simulations nor with experiments, implying that the distribution of the chain ends is greatly skewed. Although both the simulated and experimental results establish a strong correlation between *R*(*s*) and *P*(*s*), such a correlation is only valid in an ensemble sense (see Supplementary Note 14 and Supplementary Fig. 19 for additional discussions as well as ref. ^36^).

### TADs and their shapes

Our model reproduces TADs, depicted as triangles along the diagonal in Fig. 1e, of an average length of 200 kbps along the diagonal of the contact map in which the interactions between the loci are greatly enhanced. It has been noted^5^ that in a majority of cases, boundaries of the TADs are marked by a pair of CTCF motifs with a high probability of interaction between them. They are visualized as peaks in the Hi-C map (Fig. 1e). To quantitatively detect the boundaries of the TADs, we adopt the procedure described in ref. ^3^ to identify the position of each TAD (see Supplementary Note 5 for a description of the Directionality Index method for identifying TADs). The boundaries of the TADs, shown in blue (Hi-C data) and green (simulations) are reproduced by the CCM (Fig. 1e).

To investigate the sizes and shapes of each individual TADs (defined as CTCF loops in the simulations), we calculated the radii of gyration, *R*_g_, the relative shape anisotropies *κ*^2^, as well as the shape parameters, *S*, for 32 TADs (see Supplementary Note 10 for details). These TADs are typical representations of all TADs. The genomic size of the 32 TADs is similar to the genome-wide distribution (see Supplementary Fig. 2). The results are shown in Supplementary Fig. 10. The mean *R*_g_ for each individual TADs scales as their genomic length with exponent 0.27, which is an indicator of the compact structures for the TADs. However, unlike compact globular objects, their shapes are far from being globular and are much more irregular with smaller TADs adopting more irregular shapes compared to the larger TADs (see $\langle \kappa ^2\rangle$ and $\langle S\rangle$ as a function of TAD size in Supplementary Fig. 10). Such compact but irregularly shaped nature of TADs are vividly illustrated by typical snapshots for the two TADs (Fig. 1h, i). How can we understand this non-trivial highly aspherical shapes of the TADs when the chromosome is spherical on long length scales (several Mbps)? Since TADs are constrained by the CTCF loops, they may be viewed locally as ring polymers. Ring polymers in a melt are compact^37^ objects but adopt irregular shapes, consistent with our prediction for TADs.

We then wondered if TADs in each individual cells have similar sizes and shapes. We computed the dispersion in *R*_g_, *κ*, and *S* (Fig. 3c and Supplementary Figs. 10 and 11) among different trajectories. Figure3c shows the $P\left( {\langle R_{\mathrm{g}}^2\rangle /\overline {\langle R_{\mathrm{g}}^2\rangle } } \right)$, of the mean square radius of gyration $\langle R_{\mathrm{g}}^2\rangle$ for the 32 Chr 5 TADs in each trajectory normalized by the average $\overline {\langle R_{\mathrm{g}}^2\rangle }$ of each individual TAD. The bracket (bar) is the time (ensemble) average. The large dispersion in $P\left( {\langle R_{\mathrm{g}}^2\rangle /\overline {\langle R_{\mathrm{g}}^2\rangle } } \right)$ (Fig. 3c) as well as $P(\langle \kappa \rangle /\overline {\langle \kappa \rangle } )$ and $P(\langle S\rangle /\overline {\langle S\rangle } )$ (Supplementary Fig. 11) suggest that TADs are fluctuating objects, which exhibit substantial cell-to-cell variations. Our result supports the recent FISH^38^ and single-cell Hi-C experimental findings^39,40^, showing that individual TAD compaction varies widely from highly extended to compact states among different cells. To decipher how the variation of the structure of the chromosome changes as a function of *s*, we calculated the coefficient of variation, *δR*(*s*) = $\left( {\left\langle {R_s^2} \right\rangle - \left\langle {R_s} \right\rangle ^2} \right)^{1/2}/\left\langle {R\left( s \right)} \right\rangle$ (see Supplementary Note 11 for details). Interestingly, *δR*(*s*) first increases with *s* up to *s* ≈ 10^5^–10^6^ bps and then decreases as *s* further increases (Fig. 3d). Analysis of the experimental data from ref. ^6^ shows a similar decreasing trend for *s* > 10^5^ bps (Supplementary Fig. 12c). Higher resolution experiments are needed to resolve the variance for *s* < l0^5^ bps. The predicted non-monotonic dependence of *δR*(*s*) on *s* is amenable to experimental test.

### Chromosome structures in terms of the WLM

**Fig. 4:**
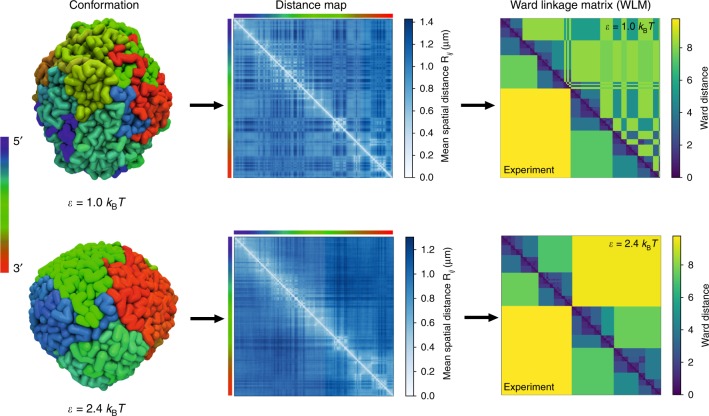
Chromosome structure in terms of ward linkage matrix (WLM). (Left) Typical conformations of the organized chromosome for $\epsilon$ = 1.0*k*_B_*T* (upper) and 2.4*k*_B_*T* (bottom). The coloring corresponds to genomic distance from one endpoint, ranging from red to green to blue. (Middle) The ensemble averaged distance maps. (Right) Comparison between the simulated WLMs (upper triangle) and the experiment WLM (lower triangle) inferred from Hi-C contact map. Ward distance is defined in the Supplementary Note 9

To quantitatively analyze the spatial organization of the compact chromosome, we use the unsupervised agglomerative clustering algorithm to reveal the hierarchy organization on the different length scales. A different method, which is also based on clustering techniques, has recently been applied to Hi-C contact map^41^. We use the WLM (see Supplementary Note 9 for details), which is directly applicable to the spatial distance matrix, **R** in which the element, *R*_*ij*_ = $\langle |{\mathbf{r}}_i - {\mathbf{r}}_j|\rangle$, is the mean spatial distance between the loci *i* and *j*. We also constructed the experimental WLM by converting the Hi-C contact map to a distance map by exploiting the approximate relationship between *R*_*ij*_ and *P*_*ij*_ $\left( { \propto R_{ij}^{ - 4.1}} \right)$ discussed previously (also see Fig. 3b). The advantages of using distance matrices instead of contact maps are two folds. First, matrix **R** is a direct depiction of the three-dimensional organization of the chromosome. The WLM, constructed from **R** is a cophenetic matrix, which can be used to reveal the hierarchical nature of the chromosome organization. Second, the contact map matrix elements do not obey triangle inequality. Therefore, it is not a good indicator of the actual 3D spatial arrangement of the loci. We show the comparison between simulated WLMs and experimentally inferred WLM for *ε*=(1.0,2.4)*k*_B_*T* (Fig. 4). Visual inspection of the WLMs for $\epsilon$ = 2.4*k*_B_*T* shows distinct segregation in the spatial arrangement of the loci. It is clear from Fig. 4 that the experimentally inferred WLM, constructed from Hi-C data, and simulations result with $\epsilon$ = 2.4*k*_B_*T* are almost identical. From the WLMs for both $\epsilon$ = 1.0*k*_B_*T* and $\epsilon$ = 2.0*k*_B_*T* (Supplementary Fig. 4), we surmise that loci with large genomic separation *s* are in spatial proximity, which is inconsistent with the experimental WLM. The Pearson correlation coefficient between experimental result and CCM using $\epsilon$ = 2.4*k*_B_*T* is 0.96 (0.53 for $\epsilon$ = 1.0*k*_B_*T*, 0.84 for $\epsilon$ = 2.0*k*_B_*T* and 0.75 for $\epsilon$ = 2.7*k*_B_*T*). Thus, the poorer agreement between the simulated WLM (Supplementary Fig. 4) as well as Spearman correlation matrix (Supplementary Fig. 6) using $\epsilon$ = (1.0, 2.0, 2.7)*k*_B_*T* and experiments, compared to $\epsilon$ = 2.4*k*_B_*T*, further justifies the latter as the optimum value in the CCM. We find it remarkable that the CCM, with only one adjusted energy scale ($\epsilon$) is sufficient to produce such a robust agreement with experiments.

### Cell-to-cell variations in the WLM

**Fig. 5:**
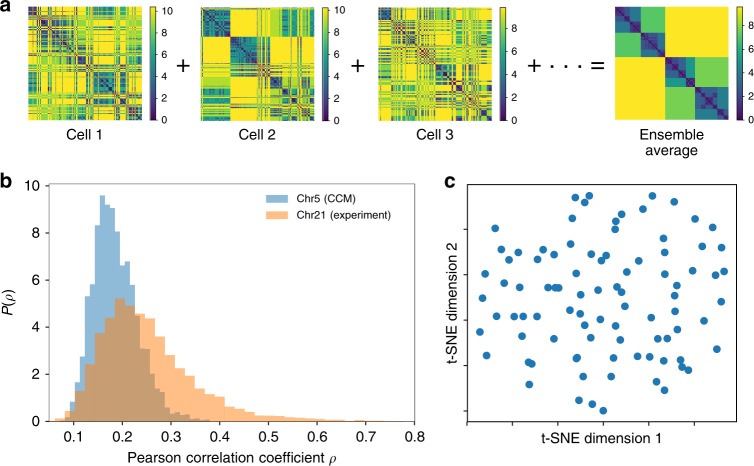
Structural heterogeneity in the chromosome. **a** Ward linkage matrices of different individual cells. The single-cell WLM is the time average result over a single trajectory. The ensemble average WLM (rightmost) and the experimental WLM are in clear quantitative agreement (Fig. 4). However, the spatial organization show large variations from cell-to-cell. Each cell has very different WLM, implying their structures are distinct. **b** The distribution of *ρ*, *P*(*ρ*), with a mean $\bar \rho$ = 0.2 (blue curve), where *ρ* is the Pearson correlation coefficient between WLMs of any two cells. The *P*(*ρ*) distribution, spanning the low range of *ρ* values, is a further demonstration of structural heterogeneity in individual cells. In yellow we plot *P*(*ρ*) with $\bar \rho _{}^{}$ = 0.25 for 120 individual human interphase Chr 21, computed using the single-cell WLMs constructed from experimental measured spatial distance data provided in ref. ^6^ **c** Two-dimensional t-SNE (t-distributed stochastic neighboring embedding) visualizations of WLM of simulated individual Chr 5 using the distance metric $\sqrt {1 - \rho }$

To assess the large structural variations between cells, we calculated the WLM for individual cells. We obtain the single-cell WLM using time averaged distance map of individual trajectories. Figure 5 shows that there are marked differences between the WLM for individual cells, with the ensemble average deviating greatly from the patterns in individual cells. Thus, the chromosome structure is highly heterogeneous. These findings are reflected in the small mean value of Pearson correlation coefficients *ρ* between all pairs of cells (Fig. 5b). The distribution *P*(*ρ*) has mean $\bar \rho$ = 0.2 with a narrow shape, implying little overlap in the WLMs between any two cells.

In order to make quantitative comparisons to experimental data, with the goal of elucidating large-scale variations in the spatial organizations of human interphase chromosomes, we constructed single-cell WLMs for Chr 21 using the spatial distance data provided in ref. ^6^ and computed the corresponding *P*(*ρ*) (Fig. 5b). The results show that the experimentally organization of Chr 21 in vivo also exhibits large variations manifested by the distribution *P*(*ρ*) covering a narrow range of low values of *ρ* with a small mean $\bar \rho$ = 0.25. Comparison to simulated result suggest that Chr 21 shows a slightly lower degree of structural heterogeneity (with a modestly larger mean $\bar \rho$ = 0.25) compared to Chr 5 investigated using CCM. Nevertheless, both the simulated and experimental results indicate that human interphase chromosomes do not have any well-defined “native structure”. To investigate whether Chr 5 has a small number of spatially distinct structures, we show two-dimensional t-SNE (t-distributed stochastic neighboring embedding) representation of 90 individual WLMs of the metric $\sqrt {1 - \rho }$ (Fig. 5c). It is clear that there is no dominant cluster, indicating that each Chr 5 in single cells is organized differently rather than belonging to a small subset of conformational states. Such large cell-to-cell variations in the structures, without a small number of well-defined states, is another hallmark of glasses, which are also revealed in recent experiments^40,42^. The presence of multiple organized structures has profound consequences on the chromosome dynamics (see below).

### Chromosome dynamics is glassy

**Fig. 6:**
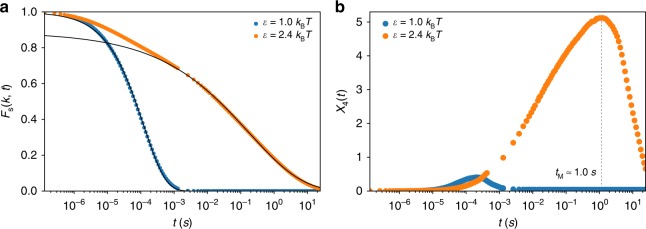
Chromosomes exhibit glassy dynamics. **a** Intermediate scattering function obtained for $\epsilon$ = 1.0*k*_B_*T* (blue) and $\epsilon$ = 2.4*k*_B_*T* (orange). The line shows an exponential function fit, *F*_s_(*k*,*t*), for $\epsilon$ = 1.0*k*_B_*T*. For $\epsilon$ = 2.4*k*_B_*T*, $F_{\mathrm{s}}(k,t) \sim e^{ - (t/t_\alpha )^\beta }$ with *β* = 0.27, for *t* exceeding a few milliseconds (black curve). **b** The fourth order susceptibility, *χ*_4_(*t*), used as a function to demonstrate dynamic heterogeneity. The peak in *χ*_4_(*t*) for $\epsilon$ = 2.4*k*_B_*T* around *t*_M_ ≈ 1 s is a signature of heterogeneity

We probe the dynamics of the organized chromosome with $\epsilon$ = 2.4*k*_B_*T*, a value that yields the best agreement with the experimental Hi-C contact map. We first calculated the incoherent scattering function, *F*_s_(*k*,*t*) = (1/*N*)$\left\langle {\mathop {\sum}\nolimits_{j = 1}^N {\mathrm{e}}^{{\mathrm{i}}{\mathbf{k}}({\mathbf{r}}_j(t) - {\mathbf{r}}_j(0))}} \right\rangle$ where ***r***_*j*_(*t*) is the position of *j*th loci at time *t*. The decay of *F*_s_(*k*,*t*) (orange line in Fig. 6a) for *k* ~ 1/*r*_s_ (*r*_s_ is the position of the first peak in the radial distribution function (*g*_AA_(*r*) and *g*_BB_(*r*)) (Fig. 2a)) is best fit using the stretched exponential function, $F_{\mathrm{s}}(k,t) \sim e^{ - (t/\tau _\alpha )^\beta }$ with a small stretching coefficient, *β* ≈ 0.27. The stretched exponential decay with small *β* is another hallmark of glassy dynamics. For comparison, *F*_s_(*k*,*t*) decays exponentially for $\epsilon$ = 1.0*k*_B_*T*, implying liquid-like dynamics (blue line in Fig. 6a).

In the context of relaxation in supercooled liquids, it has been shown that the fourth order susceptibility^43^, *χ*_4_(*k*,*t*) = $N[\langle F_{\mathrm{s}}(k,t)^2\rangle - \langle F_{\mathrm{s}}(k,t)\rangle ^2]$ provides a unique way of distinguishing between fluctuations in the liquid and frozen states. As in structural glasses, the value of *χ*_4_(*k*,*t*) increases with *t* reaching a peak at *t* = *t*_M_ and decays at longer times. The peak in the *χ*_4_(*k*,*t*) is an indication of dynamic heterogeneity, which in the chromosome is manifested as dramatic variations in the loci dynamics (see below). For $\epsilon$ = 2.4*k*_B_*T*, *χ*_4_(*k*,*t*) reaches a maximum at *t*_M_ ≈ 1 *s* (Fig. 6b), which surprisingly, is the same order of magnitude (~5 s) in which chromatin movement was found to be coherent on a length scale of ≈1 *μ*m^31^. The dynamics in *F*_s_(*k*,*t*) and *χ*_4_(*k*,*t*) together show that human interphase chromosome dynamics is glassy^27^, and highly heterogeneous. *F*_s_(*k*,*t*) and *χ*_4_(*k*,*t*) at smaller values of *k* also show that at longer length scale, chromosome exhibits glassy dynamics (Supplementary Fig. 18c, d).

### Single loci mean square displacements are heterogeneous

**Fig. 7:**
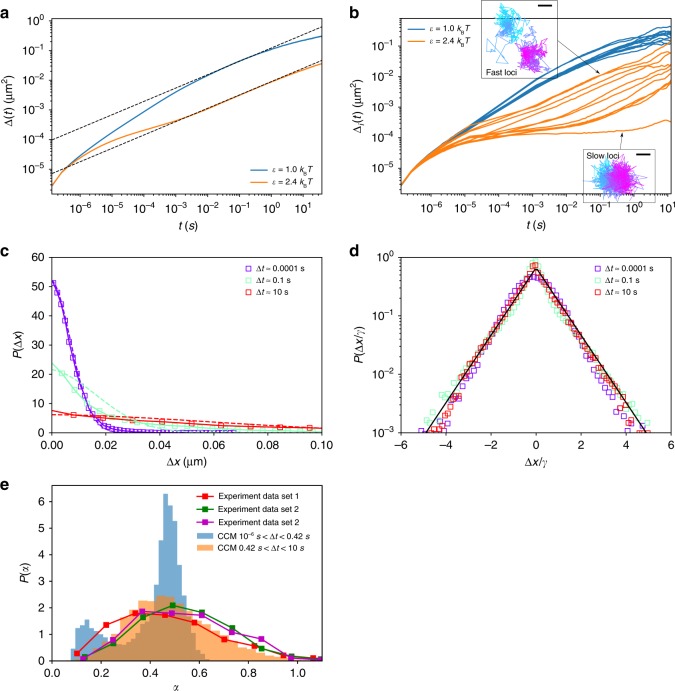
Dynamic heterogeneity of individual loci. (Top) **a** Mean square displacement, Δ(*t*), as a function of time, *t*. The effective diffusion coefficients, *D*, computed from the fitted dashed lines are 0.122 μm^2^/*t*^0.45^ and 0.009 μm^2^/*t*^0.46^ for $\epsilon$ = 1.0*k*_B_*T* and $\epsilon$ = 2.4*k*_B_*T*, respectively. **b** Time dependence of 10 single loci MSD (sMSD, Δ_*i*_(*t*)) corresponding to 1st, 1000th,..., 10,000th loci for $\epsilon$ = 1.0*k*_B_*T* and $\epsilon$ = 2.4*k*_B_*T*. The insets show Δ_*i*_(*t*) for two trajectories for fast (top) and slow (bottom) loci. Cyan (magenta) indicates short (long) lag times. The scale bar is 35 nm (0.07 nm) for fast (slow) loci. Caging effect can be clearly observed as the plateau in Δ_*i*_(*t*) for $\epsilon$ = 2.4*k*_B_*T*. **c** The Van Hove function *P*(Δ*x*) for $\epsilon$ = 2.4*k*_B_*T* at lag times Δ*t* = (0.0001,0.1,10)s. *P*(Δ*x*) has heavy tail at large Δ*x* and cannot be fit by a Gaussian (color dashed lines) except for Δ*t* = 0.0001 s at small Δ*x*. **d** Same as **c** except displacement Δ*x* is normalized by its standard deviation *γ*. *P*(Δ*x*/*γ*) for different lag times collapse onto a master curve. The black line is an exponential fit, ~*e*^−*η*(Δ*x*/*γ*)^ with *η* ≈ 1.3. **e** Distribution, *P*(*α*), of the effective diffusion exponent *α*. Comparison to experimental data^54^ are shown. The values of *α* are extracted from single loci trajectories by fitting sMSD, Δ_*i*_(*t*) ~ *t*^*α*^. The lag time range 0.42 s < Δ*t* < 10 s is in the approximate same range probed in the experiment. Experimental data set 1, 2, 3 are from Fig. 2b, c, and S5 of ref. ^54^, respectively. The results from our simulation (orange) agree well with experimental data, shown as orange. The blue bar plot is *P*(*α*) for small lag times 10^−6^ s < Δ*t* < 0.42 s. It shows two peaks, indicating the coexistence of two populations of loci with distinct mobilities

In order to ascertain the consequences of glassy dynamics at the microscopic level, we plot the MSD, Δ(*t*) = $\frac{1}{N}\left\langle {\mathop {\sum}\nolimits_{j = 1}^N (({\mathbf{r}}_j(t) - {\mathbf{r}}_{{\mathrm{com}}}(t)) - ({\mathbf{r}}_j(0) - {\mathbf{r}}_{{\mathrm{com}}}(0)))^2} \right\rangle$ in Fig. 7 where ***r***_com_ is the position of center of mass of the whole chromosome, from which a few conclusions can be drawn.

1. Because of the polymeric nature of the chromosome, the maximum excursion in $\Delta (t \to \infty ) = 2R_{\mathrm{g}}^2$, where *R*_g_ ≈ 0.7 μm is the radius of gyration of Chr 5. Consequently, for both $\epsilon$ = 1.0*k*_B_*T* and $\epsilon$ = 2.4*k*_B_T, Δ(*t*) in the long time limit is smaller than $2R_{\mathrm{g}}^2$ (Fig.7a). For $\epsilon$ = 2.4*k*_B_*T*, Δ(*t*) shows a crossover at *t* ≈ 10^−2^ s from slow to a faster diffusion, another indicator of glassy dynamics^44^. The slow diffusion is due to caging by neighboring loci, which is similar to what is typically observed in glasses. The plateau in Δ(*t*) (Fig. 7a) is not pronounced, suggesting that the compact chromosome is likely on the edge of glassiness. The crossover is more prominent in the time dependence of the mean squared displacement of single loci (see below). The slow diffusion predicted from the CCM is in accord with a number of experiments (Supplementary Fig. 21). In contrast, diffusion coefficients measured in experiments are one or two orders of magnitude smaller than the system exhibiting liquid-like behavior, which further supports the glassy dynamics for mammalian chromosomes predicted here.
2. The two dashed lines in Fig. 7a show Δ(*t*) ~ *t*^*α*^ with *α* = 0.45. The value of *α* is close to 0.4 for the condensed polymer, which can be understood using the following arguments. The total friction coefficient experienced by the whole chain is the sum of contributions from each of the *N* monomers, *ξ*_T_ = *Nξ*. The time for the chain to move a distance ≈*R*_g_ is $\tau _{\mathrm{R}} = R_{\mathrm{g}}^2/D_{\mathrm{R}} \sim N^{2{\nu} + 1}$. Let us assume that the diffusion of each monomer scales as *Dt*^*α*^. If each monomer moves a distance on the order of *R*_g_ then the chain as a whole will diffuse by *R*_g_. Thus, by equating $D\tau _{\mathrm{R}}^{\alpha} \sim R_{\mathrm{g}}^2$, we get *α* = 2*ν*/(2*ν* + 1). For an ideal chain *ν* = 0.5, which recovers the prediction by Rouse model, *α* = 0.5. For a self-avoiding chain, *ν* ≈ 0.6, we get *α* ≈ 0.54. For a condensed chain, *ν* = 1/3, we get *α* = 0.4, thus rationalizing the findings in the simulations. Similar arguments have been reported recently for dynamics associated with fractal globule^28^ and for the *β*^−^polymer model^29^. Surprisingly, *α* = 0.45 found in simulations is in good agreement with recent experimental findings^32^. We also obtained a similar result using a different chromosome model^45^, when the dynamics were examined on a longer length scale. The finding that there is no clear Rouse regime (*α* = 0.5) is also consistent with several other experimental results (Supplementary Fig. 21). We should note that distinguishing between the difference, 0.4 and 0.5, in the diffusion exponent is subtle. Additional experiments are needed to determine the accurate values of the diffusion exponents of Human interphase chromatin loci in different time regimes.
3. We also calculated the diffusion of a single locus (sMSD) defined as Δ_*i*_(*t*) = $\left\langle {({\mathbf{r}}_i(t_0 + t) - {\mathbf{r}}_i(t_0))^2} \right\rangle _{t_0}$, where $\langle \cdot \rangle _{t_0}$ is the average over the initial time *t*_0_. Distinct differences are found between the polymer exhibiting liquid-like and glassy-like dynamics. The variance in single loci MSD is large for $\epsilon$ = 2.4*k*_B_*T*, illustrated in Fig. 7b, which shows 10 typical trajectories for $\epsilon$ = 1.0*k*_B_*T* and $\epsilon$ = 2.4*k*_B_*T* each. For glassy dynamics, we found that the loci exhibiting high and low mobilities coexist in the chromosome, with orders of magnitude difference in the values of the effective diffusion coefficients, obtained by fitting Δ_*i*_(*t*) = $D_\alpha t^{\alpha _i}$. Caging effects are also evident on the time scale as long as seconds. Some loci are found to exhibit caging-hopping diffusion, which is a hallmark in glass-forming systems^46,47^. Interestingly, such caging-hopping process has been observed in human cell some time ago^48^.
4. The large variance in sMSD has been found in the motion of chromatin loci in both *E.coli* and human cells^49–53^. To further quantify heterogeneities in the loci mobilities, we calculated the Van Hove function *P*(Δ*x*), *P*(Δ*x*|Δ*t*) = $\left\langle {(1/N)\mathop {\sum}\nolimits_{i = 1}^N \delta (\Delta x - [x_i(\Delta t) - x_i(0)])} \right\rangle$. Figure 7c and d shows the *P*(Δ*x*|Δ*t*) and normalized *P*(Δ*x*/*σ*|Δ*t*) for $\epsilon$ = 2.4*k*_B_*T* at different lag times Δ*t*. For $\epsilon$ = 1.0*k*_B_*T*, Van Hove function is well fit by a Gaussian at different lag times Δ*t* (Supplementary Fig. 14). In contrast, for chromosome with glassy dynamics, all the *P*(Δ*x*|Δ*t*) exhibit fat tail, which is well fit by an exponential function at large values of Δ*x* (Fig. 7c, d) at all *δt* values, suggestive of the existence of fast and slow loci^47^.
5. The results in Fig. 7 allow us to make direct comparisons with experimental data to establish signatures of dynamic heterogeneity. We calculated the distribution of effective diffusion exponent *α*_*i*_, *P*(*α*), where *α*_*i*_ is obtained by fitting the sMSD to $\sim t^{\alpha _i}$ within some lag time (Δ*t*) range. Figure 7e shows that *P*(*α*) calculated from simulations is in good agreement with experiments^54^ in the same lag time range (0.42 s < Δ*t* < 10 s). The *P*(*α*) distribution in the range 10^−6^ s < Δ*t* < 0.42 s shows two prominent peaks, further validating the picture of coexisting fast and slow moving loci. The good agreement between the predictions of the CCM simulations with data, showing large variations of mobilities among individual loci in vivo, further supports our conclusion that organized chromosome dynamics is glassy. Interestingly, a recent computational study in which Human interphase chromosomes are modeled as a generalized Rouse chain suggests that the heterogeneity of the loci dynamics measured in live cell imaging is due to the large variation of cross-linking sites from cell-to-cell^24^. Our model implies a different mechanism that the heterogeneity observed is a manifesto of the intrinsic glassy dynamics of chromosomes.

### Active loci has higher mobility

**Fig. 8:**
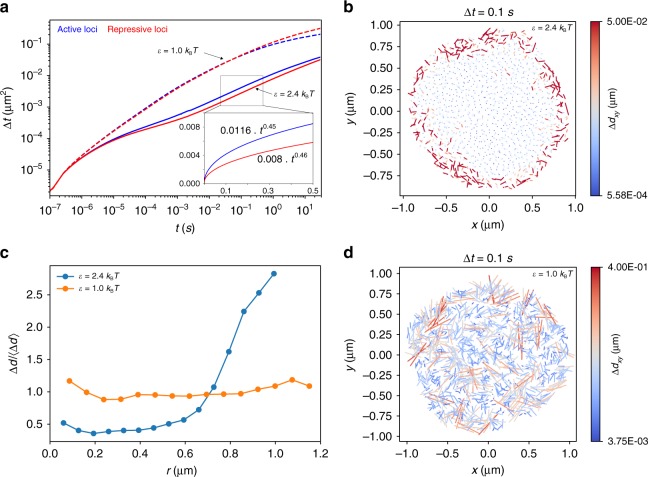
Mobility of active and repressive loci. **a** The mean square displacement for active loci and repressive loci. The equation shown in the inset is the fit using *Dt*^*α*^, where *D* is the diffusion coefficient and *α* is the diffusion exponent. **b** The displacement vectors of the loci within the equator cross-section of the structured chromosome for $\epsilon$ = 2.4*k*_B_*T*. The displacements are computed for time window Δ*t* = 0.1 s. The color bars on the right show the magnitudes of the displacements. **c** Displacement Δ*d* normalized by its mean as a function of radial position, *r*, of the loci. **d** Same as **b** except the results are obtained using $\epsilon$ = 1.0*k*_B_*T*

Figure 8a shows MSD for active and repressive loci. For $\epsilon$ = 1.0*k*_B_*T*, there is no difference between active and repressive loci in their mobilities. However, in the glassy state active loci diffuses faster than the repressive loci. The ratio between the effective diffusion coefficients (the slope of the dashed line) of the active and repressive loci is 0.0116/0.008 $\simeq$ 1.45, in good agreement with experimental estimate 0.018/0.013 $\simeq$ 1.38^32^. Such a difference in their mobilities is also confirmed by *F*_s_(*k*,*t*) and *χ*_4_(*t*) (Supplementary Note 13 and Supplementary Fig. 18a, b). These variations are surprising since the parameters characterizing the A–A and B–B interactions are identical. To investigate the origin of the differences between the dynamics of A and B loci, we plot the displacement vectors of the loci across the cross-section of the condensed chromosome (Fig. 8b) for a time window Δ*t* = 0.1 s. The loci on the periphery have much greater mobility compared to the ones in the interior. In sharp contrast, the fluid-like state exhibits no such difference in the mobilities of A and B (Fig. 8d). To quantify the dependence of the mobility on the radial position of the loci, we computed the amplitude of the displacement normalized by its mean, as a function of the radial position of the loci, *r* (Fig. 8c). For the chromosome exhibiting glass-like behavior, the mobility increases sharply around *r* ≈ 0.7 μm whereas it hardly changes over the entire range of *r* in the fluid-like system. Because the active loci are mostly localized on the periphery and the repressive loci are in the interior (Fig. 2b), the results in Fig. 8 suggest that the differences in the mobilities of the loci with different epigenetic states are due to their preferred locations in the chromosome. It is intriguing that glassy behavior is accompanied by a position-dependent mobility, which can be understood by noting that the loci in the interior are more caged by the neighbors, thus restricting their movement. In a fluid-like system, the cages are so short-lived that the apparent differences in the environments the loci experience are averaged out on short timescales. Note that in the experimental result^32^ comparison is made between the loci in the periphery and interior of the nucleus. It is well known that the nucleus periphery is enriched with heterochromatin (repressive loci) and the interior is enriched with euchromatin (active loci). However, for an individual chromosome, single-cell Hi-C study^40^ and other experimental studies^55–58^ suggest that the active loci are preferentially localized at the surface of the chromosome territory.

## Discussion

In order to demonstrate the transferability of the CCM, we simulated Chr 10 using exactly the same parameters as for Chr 5 (Supplementary Note 12). Supplementary Fig. 16 compares the WLM obtained from simulations for different $\epsilon$ values and the computed WLM using the Hi-C contact map. The contact map is translated to the distance *R*_*ij*_ by assuming that $P_{ij} \propto R_{ij}^{ - 4.1}$ holds for Chr 10 as well. It is evident that the CCM nearly quantitatively reproduces the spatial organization of Chr 10 (Supplementary Fig. 16). Thus, it appears that the CCM could be used for simulating the structures and dynamics of other chromosomes as well.

**Fig. 9:**
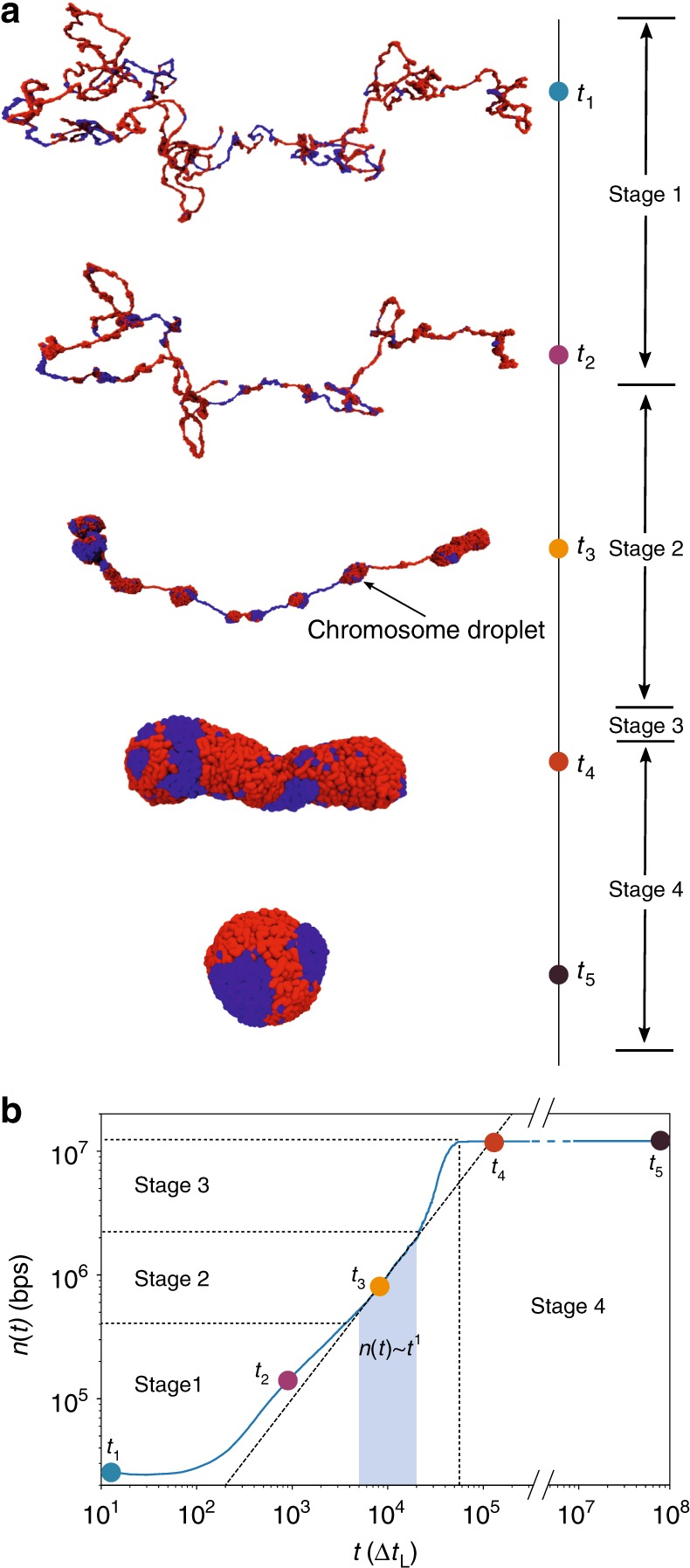
Dynamics of chromosome organization. **a** Typical conformations sampled during the chromosome organization process. After the short initial folding process (stage 1, *t*_1_ and *t*_2_), the chromosome droplets (CDs) connected by “tension strings” begin to form (stage 2, *t*_3_). The average size of CDs at the onset of CD formation is about *s* ~ 4 × 10^5^ bps, which coincides with approximate value of s*, the typical size of TADs (Fig. 1b). At the later stage (stage 3, conformation not shown here), CDs merge to form larger cluster, eventually form the final condensed structure (stage 4, *t*_4_ and *t*_5_). Red (blue) represents repressive (active) loci. **b** The time-dependent growth of CDs, *n*(*t*), which is the average number of base pairs in a CD at *t*. The dashed line is a fit in the time window indicated by the shaded area, yielding *n*(*t*) ~ *t*^1^. The roughly linear increase of *n*(*t*), over a range of times, is consistent with the Lifshitz–Slazov growth mechanism^59^. For a vivid demonstration, see Supplementary Movie 1

Two scaling regimes in *P*(*s*) is suggestive of scale-dependent folding of genome. In order to reveal how chromosome organizes itself and to link these processes to the experimentally measurable *P*(*s*), we calculated the time-dependent change in *P*(*s*) as a function of *t*. At scales less (above) than s* ≈ 5×10^5^bps, *P*(*s*) decreases (increases) as the chromosome becomes compact. The *P*(*s*) ~ *s*^−0.75^ scaling for *s* < s* (see also Fig. 1b) is the result of organization on the small genomic scale during the early stage of chromosome condensation (Fig. 9a). In the initial stages, compaction starts by forming ≈s* sized chromosome droplets (CDs) as illustrated in Fig. 9a. In the second scaling regime, *P*(*s*) ~ *s*^−1.25^, global organization occurs by coalescence of the CDs (Fig. 9a). Thus, our CCM model, which suggests a hierarchical chromosome organization on two distinct scales, also explains the two scaling in *P*(*s*).

The hierarchical nature of the structural organization is further illustrated using *A*(*s*), the number of contacts that a *s*-sized subchain forms with the rest of the chromosome. For a compact structure, *A*(*s*) ~ *s*^2/3^ and *A*(*s*) ~ *s* for an ideal chain. Supplementary Fig. 17 shows that *A*(*s*) computed using the Hi-C data (black square line) varies as *s*^2/3^, suggesting that chromosome is compact on all length scales. We also find that upon increasing *ε*, the range of *A*(*s*) ~ *s*^2/3^ expands. The pictorial view of chromosome organization (Fig. 9a) and the *A*(*s*) scaling show that chromosome structuring occurs hierarchically with the formation of CDs and subsequent growth of the large CDs at the expense of smaller ones. We quantitatively monitored the growth of CDs during the condensation process and found that the size of CD grows linearly with time during the intermediate stage (Fig. 9b). Such a condensation process is reminiscent of the Lifshitz–Slazov mechanism^59^ used to describe Ostwald ripening.

Our simulations show that the average TAD size and the crossover scale (s*) the dependence of *P*(*s*) on *s* coincide. In addition, the size of the CDs is also on the order of s*, which is nearly the same for all the chromosomes (Fig. 1c). We believe that this is a major result. The coincidence of these scales suggests that both from the structural and dynamical perspective, chromosome organization takes place by formation of TADs, which subsequently arrange to form structures on larger length scales. Because gene regulation is likely controlled by the TADs, it makes sense that they are highly dynamic. We hasten to add that the casual connection between TAD size and s* as well as the CDs size has to be studied further. If this picture is correct then chromosome organization, at length scales exceeding about 100 kbps, may be easy to describe.

In summary, we developed the CCM, a self-avoiding polymer with two epigenetic states and with fixed loop anchors whose locations are obtained from experiment to describe chromosome dynamics. The use of rigorous clustering techniques allowed us to demonstrate that the CCM nearly quantitatively reproduces Hi-C contact maps, and the spatial organization gleaned from super-resolution imaging experiments. It should be borne in mind that contact maps are probabilistic matrices that are a low dimensional representation of the three-dimensional organization of genomes. Consequently, many distinct copolymer models are likely to reproduce the probability maps encoded in the Hi-C data. In other words, solving the inverse problem of going from contact maps to an energy function is not unique^9^.

Chromosome dynamics is glassy, with correlated dynamics on scale ≈ 1 μm, implying that the free energy landscape has multiple equivalent minima. Consequently, it is likely that in genomes only the probability of realizing these minima is meaningful, which is the case in structural glasses. The presence of multiple minima also leads to cell-to-cell heterogeneity with each cell exploring different local minimum in the free energy landscape. We speculate that the glass-like landscape might also be beneficial in chromosome functions because only a region on size ~s* needs to be accessed to carry out a specific function, which minimizes large-scale structural fluctuations. In this sense, chromosome glassiness provides a balance between genomic conformational stability and mobility.

## Methods

### Construction of the CCM

Contact maps^4,5^ of interphase chromosomes show that they are partitioned into genome-wide compartments, displaying plaid (checkerboard) patterns. If two loci belong to the same compartment they have the higher probability to be in contact than if they are in different compartments. Although finer classifications are possible, compartments^4^ can be categorized broadly into two (open (A) and closed (B)) classes associated with distinct histone markers. Open compartment is enriched with transcriptional activity-related histone markers, such as H3K36me3, whereas the closed compartment is enriched with repressive histone markers, such as H3K9me3. Chromatin segments with repressive histone markers have effective attractive interactions, which models HP1 protein-regulated interactions between heterochromatin regions^60,61^. We assume that chromatin fiber, with active histone markers, also has such a similar attraction. From these considerations, it follows that the minimal model for human chromosome should be a copolymer where the two types of monomers represent active and repressive chromatin states. To account for the two states, we introduce the CCM as a self-avoiding polymer with two kinds of beads. Similar genre of models have been proposed in several recent studies^18–21,26^ to successfully decipher the organization of genomes.

The energy function in the CCM is, *E* = *U*_C_ + *U*_LJ_, where *U*_C_ contains bond potential (*U*^S^) and loop interaction (*U*^L^), and *U*_LJ_ is the Lennard–Jones pairwise interaction between the monomers (see Supplementary Note 1 for details). If two monomers belong to type A (B), the interaction strength is $$\epsilon _{{\mathrm{AA}}}$$$$\left( {\epsilon _{{\mathrm{BB}}}} \right)$$. The interaction strength between A and B is $$\epsilon _{{\mathrm{AB}}}$$. Each monomer represents 1200 base pairs (bps), with six nucleosomes connected by six linker DNA segments. The size of each monomer, *σ*, is estimated by considering two limiting cases. If we assume that nucleosomes are compact then the value of *σ* may be obtained by equating the volume of the monomer to 6*v* where *v* is the volume of a single nucleosome. This leads to *σ* ≈ 6^1/3^*R*_N_ ≈ 20 nm where *R*_N_ ≈ 10 nm is the size of each nucleosome^62^. Another limiting case may be considered by treating the six nucleosome array as a worm-like chain. The persistence length of the chromatin fiber is estimated to be ~1000 bps^63^, which is about the size of one monomer. The mean end-to-end distance of a worm-like chain whose persistence length is comparable to the contour length *L* is $$R \approx L\sqrt {2/e}$$. The value of *L* for a six nucleosome array is 6(16.5 + *R*_N_)nm where the length of a single linker DNA is 16.5 nm. This gives us the upper bound of *σ* to be 130 nm. Thus, the two limiting values of *σ* are 20 nm and 130 nm. We assume that the value of *σ* is an approximate mean, yielding *σ* = 70 nm.

The type of monomer is determined using the Broad ChromHMM track^64^. There are totally 15 chromatin states, out of which the first eleven are related to gene activity. Thus, we consider state 1 to state 11 as a single active state (A) and states 12–15 as a single repressive state (B). For the genome range 146–158 Mbps in the Chromosome 5 in Human GM12878 cell, which is investigated mainly in this work, the numbers of active and repressive loci are 2369 and 7631, respectively. The positions of the loop anchors in CCM are determined from Hi-C experiment^5^. Details of the assignment of monomer type and loop anchors are given in the Supplementary Note 2.

### Simulations

As detailed in the Supplementary Notes 3 and 4, we performed simulations using both Langevin dynamics (low friction) and Brownian dynamics (high friction) using a custom-modified version of the molecular dynamics package LAMMPS. The use of Langevin dynamics accelerates the sampling of the conformational space^65^, needed for reliable computation of static properties. Realistic value of the friction coefficient is used in Brownian dynamics simulations to investigate chromosome dynamics, thus allowing us to make direct comparisons with experiments.

We varied the values of $\epsilon _{{\mathrm{AA}}}$, $\epsilon _{{\mathrm{BB}}}$ and $\epsilon _{{\mathrm{AB}}}$ to investigate the effect of interaction strength on the simulation results. For simplicity, we set $\epsilon _{{\mathrm{AA}}} = \epsilon _{{\mathrm{BB}}} \equiv \epsilon$. By fixing the ratio $\epsilon {\mathrm{/}}\epsilon _{{\mathrm{AB}}}$ to 11/9, $\epsilon$ is the only relevant energy scale in the CCM. The results presented in the main text are obtained with $\epsilon$ = 2.4*k*_B_*T* unless stated otherwise. The contacts between loci in the simulation are determined by the threshold distance *r*_c_ = 2*σ* where *σ* = 70 nm. We should note that by independently tuning three parameters $\epsilon _{{\mathrm{AA}}}$, $\epsilon _{{\mathrm{BB}}}$, and $\epsilon _{{\mathrm{AB}}}$ separately, the model could be further optimized in the comparison with experiment Hi-C data. For the simplicity and because the errors in Hi-C data are hard to quantify, we make the assumption that $\epsilon _{{\mathrm{AA}}} = \epsilon _{{\mathrm{BB}}} \equiv \epsilon$ and fix the ratio $\epsilon _{{\mathrm{AB}}}{\mathrm{/}}\epsilon$, resulting in only one free parameter, either $\epsilon _{{\mathrm{AB}}}$ or $\epsilon$. In practice, we varied $\epsilon$ while keeping the ratio $\epsilon _{{\mathrm{AB}}}{\mathrm{/}}\epsilon$ a constant. Our results suggest that even with this simplification, the results based on the CCM produces near quantitative agreement with Hi-C data.

The folding of chromatin is simulated starting from extended conformations (Supplementary Note 4). Due to the slow relaxation process, theoretically predicted in a previous study^27^, and topological constraints^14^, long polymers such as human interphase chromosomes are unlikely to come to equilibrium even on the time scale of a single-cell cycle. Thus, the initial conformations could in principle affect the organization of genomes. Although the folding from an extended conformation is unlikely to occur for chromosome as a whole in vivo, we believe that the folding process investigated in this work provides insights into gene activation because it involves only local folding or unfolding^66–68^.

### Data availability

The data that support the findings of this study are available from the authors upon reasonable request.

### Code availability

The custom LAMMPS code used in this study for BD simulations are provided in the public GitHub repository https://github.com/anyuzx/Lammps_brownian.

## Electronic supplementary material

Supplementary Information

Description of Additional Supplementary Files

Supplementary Movie 1
