# Supplementary material for: Interphase human chromosome exhibits out of equilibrium glassy dynamics
Source: Nat Commun. 2018 Aug 8;9:3161. doi: 10.1038/s41467-018-05606-6 (PMC6082855; doi:10.1038/s41467-018-05606-6)
Supplement: Supplementary file 1 — Supplementary Information [file 41467_2018_5606_MOESM1_ESM.pdf]

Supplementary Information: Interphase Human Chromosome Exhibits Out of Equilibrium Glassy Dynamics

Guang Shi[1], Lei Liu[2], Changbong Hyeon[2] and D. Thirumalai[3*]

[1]*Biophysics Program,*

Institute for Physical Science and Technology,

University of Maryland,

College Park, MD 20742

[2]*Korea Institute for Advanced Study,*

Seoul 02455, Republic of Korea

[3]*Department of Chemistry,*

University of Texas at Austin, Texas, 78712

* Corresponding author: dave.thirumalai@gmail.com

Supplementary Note 1: Model

For reasons explained in the main text, we model the interphase chromosome as a self-avoiding AB-copolymer with A (B) type beads representing the active (repressive) chromatin (Supplementary Fig.1 and Fig.1a in the main text). Note that in many of the polymer models developed to reproduce Hi-C contact maps, self-avoidance is not strictly enforced, which is partially justified because Topoisomerase facilitates chain crossing. We do not find it necessary to impose this restriction. The chromosome copolymer model (CCM) potential energy is,

$$U(\mathbf{r}_1, ..., \mathbf{r}_N) = \sum_{i=1}^{N-1} U_i^{\mathrm{S}} + \sum_{i=1}^{N-1} \sum_{j=i+1}^{N} U_{i,j}^{\mathrm{P}} + \sum_{\{p,q\}} U_{\{p,q\}}^{\mathrm{L}} \tag{A1}$$

For the bond stretch potential, U_i^s, we use the FENE (Finite Extensible Nonlinear Elastic) potential given by,

$$U_i^{\mathrm{S}} = -\frac{1}{2} K_{\mathrm{S}} R_0^2 \ln\Big[1 - \Big(\frac{|\mathbf{r}_{i+1} - \mathbf{r}_i|}{R_0}\Big)^2\Big] \tag{A2}$$

where R_0 is the equilibrium bond length, and K_{S} is the spring constant.

The interaction between beads accounting for steric repulsion and attraction is given by the Lennard-Jones potential with different parameters for the distinct bead types. The potential between the active locus and repressive locus is,

$$U_{i,j}^{\mathrm{P}} \equiv U_{\alpha\beta}(r = |\mathbf{r}_i - \mathbf{r}_j|) = 4\epsilon_{\alpha\beta}\Big[\Big(\frac{\sigma}{r}\Big)^{12} - \Big(\frac{\sigma}{r}\Big)^{6}\Big] \tag{A3}$$

where α and β can be either A (active/euchromatin) or B (repressive/heterochromatin). For simplicity, we assume that the σ value for the active state (A) and the repressive state (B) is identical.

The interaction between the loop anchors is modeled using a harmonic potential,

$$U_{\{p,q\}}^{\mathrm{L}} = K_{\mathrm{L}}(|\mathbf{r}_p - \mathbf{r}_q| - a)^2 \tag{A4}$$

where $\{p, q\}$ is the index of the loop, and a is the equilibrium bond length between the loop pairs. The indices of loop anchors, modeling the role of CTCF motifs, taken from the Hi-C data [1], are listed in Table I. The values of all the parameters in the CCM model of the chromosome are given in Table II.

The virtue of the CCM is that it has essentially only one energy scale ϵ given that we have assumed that $\epsilon_{AA} = \epsilon_{BB}$. Explicit simulations show that this is sufficient to capture not only the compartments and TADs in the contact map reasonably well but also the chromosome dynamics. The inclusion of other epigenetic states identified in experiments may produce better agreement with the contact map inferred from Hi-C experiment but comes at the expense of introducing additional parameters. It is unlikely that such a model would alter the chromosome dynamics, which is the focus of model study.

In the main text, we made references to copolymer models, which have been previously used to study chromatin organization. The one that is most similar to CCM is the block copolymer model used to describe the architecture of the roughly one Mbps *Drosophila* genome [2]. In their model micro-phase separation results by adjusting a non-specific energy scale between all loci pairs to induce global chain compaction and specific interaction (the analog of ϵ_{AA}, ϵ_{BB} and ϵ_{AB} in the CCM) between identical epigenetic states. A related minimal model, with three epigenetic states, was recently introduced in [3] that accounts for active, inactive, and unmarked states. These models, along with CCM, show that many aspects of chromosome organization can be captured using a minimum number of free parameters.

With the assumption that $\epsilon_{AA} = \epsilon_{BB} = \epsilon$, the only free parameter in the CCM is ϵ_{AB}. The only physical requirement for choosing a specific value of ϵ_{AB} is that loci with distinct epigenetic state should segregate in order to capture the compartment feature that is prominent in the Hi-C maps. For the interaction parameter values listed in the third row of Table II, which is most appropriate for interphase chromosomes 5 and 10, loci A and B do not mix. In other words they phase separate. This can be rationalized by adopting a Flory-Huggins type argument, which involves calculating the second virial coefficient, $B_{2,\alpha\beta} = 2\pi \int \mathrm{d}r r^2 [1 - e^{-U_{\alpha\beta}/(k_{\mathrm{B}}T)}]$. We find that for the parameters in the third row of Table 2, $|B_{2,AB}| < |B_{2_A A}|$, which implies that A and B loci tend not to mix. Note that $B_{2_A A} = B_{2_B B}$ in the CCM. This argument shows that for any value of ϵ_{AB} for which the inequality $|B_{2,AB}| < |B_{2_A A}|$ is satisfied, the copolymer would exhibit microphase separation. However, the extent of segregation will depend on the precise numerical

values. For our energy function, the values listed in third row of Table II is optimal, because simulations using them provide the best agreement with the measured Hi-C contact maps.

Supplementary Note 2: Identification of the monomer type and loop anchors from experimental data

The epigenetic state of each bead is determined using the Broad ChromHMM track [4–6]. There are a total of 15 chromatin states in the track. For simplicity, we assign states 1-11 to be in the active state (A) and states 12-15 to be in the repressive state (B). This is reasonable since all the states between 1 and 11 are related to gene transcription, and hence can be modeled as euchromatin. States 12 to 15 are polycomb repressed, heterochromatin or repetitive region, which we modeled as heterochromatin. ChromHMM track has a resolution of 200bps which is smaller than 1,200bps representing one monomer in the CCM. We first count the number of basepairs of state A and B within the 1,200 basepairs segment represented by each monomer. Then the type of each monomer is assigned as the state with a larger number of basepairs. Such a coarse-graining procedure may not be appropriate when the number of bps of the two types has a similar value in the 1,200bps segment. Although this is a possible outcome, we found that most of the 1,200bps long segments are overwhelmingly occupied by only one state, corresponding to either active or repressive state. For loop anchors, we directly used the Hi-C data [1]. The locations of loops are provided in the file *GSE63525_GM12878_primary+replicate_HiCCUPS_looplist_with_motifs.txt.gz* under the GEO accession number GSE63525. We only selected the loops with CTCF motifs "uniquely" called at both anchors (see Section VI.e.7 of the Extended Experimental Procedures of [1]). For each pair of CTCF loop anchors, we assign a harmonic constraint (Eq. A4) between the two corresponding loci.

Supplementary Note 3: Simulation details

We use both low friction Langevin Dynamics (LD) and Brownian dynamics (BD) to simulate

the equilibrium and dynamical properties of the chromosome. The equation of motion for the ith locus is given by,

$$m_i\ddot{\mathbf{r}}_i = \mathbf{F}_i - \xi\dot{\mathbf{r}}_i + \mathbf{R}_i(t), \tag{B1}$$

where ξ is the friction coefficient, $\mathbf{F}_i$ is the systematic force $-\frac{\partial U}{\partial \mathbf{r}_i}$ experienced by each bead, and $\mathbf{R}_i(t)$ is the random force mimicking the thermal fluctuation of the surrounding environment. In Eq. (B1), $\mathbf{R}_i(t)$ is the Gaussian random force that satifies the fluctuation-dissipation theorem $\langle R_i\rangle = 0$ and $\langle \mathbf{R}_i(t)\cdot\mathbf{R}_j(t^{'})\rangle = 6k_{\mathrm{B}}T\xi\delta(t-t^{'})\delta_{ij}$. The LD simulations are performed using the Molecular Dynamics software LAMMPS [7, 8] in which the equation of motion are integrated using the velocity-Verlet algorithm. The sampling of the conformations are accelerated in LD, as was shown previously [9], and hence, we use LD simulations to generate well-equilibrated conformations.

The equation of motion for BD, derived by neglecting the inertial term in Eq. (B1), is,

$$\dot{\mathbf{r}}_i = \frac{1}{\xi}\mathbf{F}_i + \frac{1}{\xi}\mathbf{R}_i(t). \tag{B2}$$

We modified the LAMMPS software to perform the BD simulations, thus allowing us to obtain a realistic description of the dynamics. The use of BD also allows us to calculate the timescales for the chromosome dynamics, which can be directly compared to experiments. We employed the Euler algorithm to integrate the equation of motion in Eq. (B2).

For BD, the relevant time scale is $\tau_{\mathrm{B}} = \sigma^2/D$ where $D = kT/\xi$, and $\xi = 6\pi\eta\sigma/2$. We choose our integration time step to be $\Delta t_{\mathrm{B}} = 0.0001\tau_{\mathrm{B}}$. With the choice of σ = 70nm, we obtain $D = \frac{kT}{6\pi\eta\sigma/2} \approx 7.0\mu m^2/s$ with $\eta = 0.89\times 10^{-3} Pa\cdot s$. Thus, the value of $\tau_{\mathrm{B}} \approx 0.0007s$. For the LD simulations, we use the time step $\Delta t_{\mathrm{L}} = 0.01\tau_{\mathrm{L}}$ where $\tau_{\mathrm{L}} = \sqrt{m\sigma^2/kT}$.

Supplementary Note 4: Generation of the initial conformations and production runs

The copolymer is initially prepared as a rod. After determining the positions of the loop anchors, we performed simulations using LD with temperature $T = 1.0$ (measured in the unit of $k_{\mathrm{B}}T$) using the WCA potential with the same parameter values regardless of the bead type. We used

the WCA potential,

$$U_{\alpha\beta}(r = |\mathbf{r}_i - \mathbf{r}_j|) = \begin{cases} 4\epsilon\left[\left(\frac{\sigma}{r}\right)^{12} - \left(\frac{\sigma}{r}\right)^{6}\right] + \epsilon, & \text{if } 0 < r < 2^{1/6}\sigma. \\ 0, & \text{otherwise.} \end{cases} \tag{C1}$$

with $\epsilon = 1.0k_BT$ and $\sigma = 1$. Since all the loop anchor pairs initially are spatially well-separated, we first performed simulations using a small time step ($\Delta t_L = 10^{-6}\tau_{\mathrm{L}}$) to avoid numerical instabilities. After a certain number of time steps, all the loop pair beads are in proximity fluctuating around their equilibrium bond distance. At this stage, we increased the value of the time step to $\Delta t_{\mathrm{L}} = 0.01\tau_{\mathrm{L}}$, and turned on the attractive pairwise interaction (Eq.A3), and continued the simulations for an additional $10^8\Delta t_{\mathrm{L}}$. We monitored the radius of gyration, R_g, to ensure that R_g fluctuates around a mean value as one indication of thermalization (Supplementary Fig.3a). In addition, the potential energy (Supplementary Fig.3b) has reached plateau values, which is a necessary condition indicating that the copolymer has adequately sampled a large number of distinct conformations. We also computed the evolution of $P(s)$ during the pre-production run (Supplementary Fig.5). The negligible change in $P(s)$ also suggest convergence of our simulations from the perspective of structural measures. We then performed LD simulations for an additional $10^8\Delta t_{\mathrm{L}}$ to compute the static structural properties. The final chain conformations obtained at the end of production runs are used as initial conformations in the subsequent BD simulations.

Supplementary Note 5: Determination of TADs boundaries using the Directionality Index and Hidden Markov Model

We adopt the methods first introducted in [10] to determine TAD boundaries. The Directionality Index (DI) for the ith loci is computed using,

$$\mathrm{DI}_i = \left(\frac{B_i - A_i}{|B_i - A_i|}\right)\left(\frac{(A_i - E_i)^2}{E_i} + \frac{(B_i - E_i)^2}{E_i}\right), \tag{D1}$$

where A_i, B_i are, respectively, the number of contacts the ith locus forms with one that is L bps upstream and the number of contacts from the same locus to the one L bps downstream, and

$E_i = (A_i + B_i)/2$. We calculated A_i and B_i using $A_i = \sum_{j=i+1}^{j=i+L} C_{ij}$ and $B_i = \sum_{j=i-L}^{j=i-1} C_{ij}$ where C_{ij} is the number of contacts between i^{th} and j^{th} loci. The results shown in Fig.1e in the main text are computed using $L = 375$ kbps.

The boundaries of the TADs are then determined by a Hidden Markov Model (HMM) with Gaussian emissions. HMM takes the $\mathrm{DI_i}$ as input, and produces an output which is the inferred hidden state. Unlike in the study by Dixon et al [10], we use only two states. The boundaries of each TADs are the loci where the hidden states change. For instance, suppose we have hidden states $(-1, -1, -1, 1, 1, 1, 1, -1, -1)$ where the ith element represents the hidden state of ith loci, then there are three TADs and the location of the boundaries of each TAD would be $(1, 3)$,$(4, 7)$ and $(8, 9)$.

Supplementary Note 6: Approximate equivalence of the distance between the loci and centroid distance between TADs

In Ref. [11], the spatial distance is measured between the centroid locations of a pair of TADs, which can be used to extract information about TAD structures. Let us consider two TADs labeled k and l. We denote the radii of gyration of the two TADs as R_k and R_l and the centroids as $\mathbf{C}_k$ and $\mathbf{C}_l$. We show here that under the condition that sizes of the TADs are small compared to the distance between the two centroids ($R_k + R_l \ll |\mathbf{C}_k - \mathbf{C}_l|$), the spatial distance measured in the simulations is equivalent to the distance between TADs inferred from the imaging experiment. More precisely, $(\mathbf{C}_k - \mathbf{C}_l)^2 \approx \overline{(\mathbf{r}^{(k)} - \mathbf{r}^{(l)})^2}$, where $\mathbf{r}^{(k)}$ and $\mathbf{r}^{(l)}$ are the positions of the loci in the kth and the lth TADs. If N_k and N_l are the genomic sizes of the two TADs, then the average square of the distance between two loci belonging to the kth and the lth TADs is,

$$\overline{(\mathbf{r}^{(k)} - \mathbf{r}^{(l)})^2} \equiv \frac{1}{N_k N_l} \sum_i \sum_j (\mathbf{r}_i^{(k)} - \mathbf{r}_j^{(l)})^2, \qquad \text{(E1)}$$

where $\mathbf{r}_i^{(k)}$ and $\mathbf{r}_j^{(l)}$ are the ith loci in the kth TAD and the jth loci in the lth TAD, respectively.

Expansion of the the right hand side of Eq. (E1) gives,

$$
\begin{aligned}
\overline{(\mathbf{r}^{(k)} - \mathbf{r}^{(l)})^2} &= \frac{1}{N_k}\sum_{i=1}^{N_k} \mathbf{r}_i^{(k)^2} + \frac{1}{N_l}\sum_{j=1}^{N_l} \mathbf{r}_j^{(l)^2} - 2\mathbf{C}_k\mathbf{C}_l \\
&= \frac{1}{N_k}\sum_{i=1}^{N_k} \mathbf{r}_i^{(k)^2} + \frac{1}{N_l}\sum_{j=1}^{N_l} \mathbf{r}_j^{(l)^2} - \mathbf{C}_i^2 - \mathbf{C}_j^2 + (\mathbf{C}_k - \mathbf{C}_l)^2 \\
&= \frac{1}{N_k}\sum_{i=1}^{N_k} (\mathbf{r}_i^{(k)} - \mathbf{C}_k)^2 + \frac{1}{N_l}\sum_{j=1}^{N_l} (\mathbf{r}_j^{(l)} - \mathbf{C}_l)^2 + (\mathbf{C}_k - \mathbf{C}_l)^2 \\
&= R_k^2 + R_l^2 + (\mathbf{C}_k - \mathbf{C}_l)^2 \approx (\mathbf{C}_k - \mathbf{C}_l)^2,
\end{aligned}
\tag{E2}
$$

where the last step is a result of the assumption that $R_k + R_l \ll |\mathbf{C}_k - \mathbf{C}_l|$. Thus, the monomer-monomer spatial distance computed from simulations is approximately equivalent to the experimental measurement of the distance between the TADs. The good correlation between experiment and simulation (in Fig.3a in the main text) justifies our assumption.

Supplementary Note 7: Spearman correlation map

In order to quantitatively assess the closeness of the simulated and experimental contact maps, we first calculated the Spearman correlation maps. When computing the Spearman correlation map, the contact map obtained from our simulations or the Hi-C data is first transformed to a log scale. For each entry, c_{ij}, in the transformed log scale contact map, we calculated the Z-Score using $z_{ij} = (c_{ij} - \langle c_s \rangle)/\sigma_s$ where $\langle c_s \rangle = (1/(N-s))\sum_{i<j}\delta(s-(j-i))c_{ij}$, and σ_s is the standard deviation of c_s. The Spearman correlation coefficient, ρ_{ij}, is calculated between the ith row, $\mathbf{X}_i$, and the jth column, $\mathbf{Y}_j$, of the matrix $\mathbf{Z}$ whose elements are z_{ij}. The Spearman correlation coefficient is defined as the Pearson correlation coefficient between the ranked variables. First, the raw vector $\mathbf{X}_i$ and $\mathbf{Y}_j$ are converted to rank variable $\mathbf{R}_{X_i}$ and $\mathbf{R}_{Y_j}$ by assigning a rank of 1 to the lowest value in the $\mathbf{R}_{X_i}$ and $\mathbf{R}_{Y_j}$ vectors, and 2 to the next lowest and so on. The Spearman correlation coefficient is the Pearson correlation coefficient between two rank variable vectors $\mathbf{R}_{X_i}$ and $\mathbf{R}_{Y_j}$, computed using $\rho_{ij} = \text{cov}(\mathbf{R}_{X_i}, \mathbf{R}_{Y_j})/(\sigma_{\mathbf{R}_{X_i}}\sigma_{\mathbf{R}_{Y_j}})$ where $\text{cov}(\mathbf{R}_{X_i}, \mathbf{R}_{Y_j})$ is the covariance between $\mathbf{R}_{X_i}$ and $\mathbf{R}_{Y_j}$ and $\sigma_{\mathbf{R}_{X_i}}$,$\sigma_{\mathbf{R}_{Y_j}}$ are the standard deviation of $\mathbf{R}_{X_i}$ and $\mathbf{R}_{Y_j}$. The elements in the Spearman correlation matrix, ρ_{ij}, are the Spearman

correlation coefficients between $\mathbf{X_i}$ and $\mathbf{Y_j}$.

Supplementary Note 8: Comparison of the Correlation Maps

We use quantitative measures to assess if the simulated and experimentally inferred contact and correlation maps are similar. In particular, we compare as precisely as possible, the compartment patterns suggested in the Hi-C map and the simulation results from the CCM. For a fixed genomic distance, the contact probability between two loci of the same type is greater than between two loci of different types. The task is to partition the loci based on the contact map such that each partition corresponds to one distinct loci type while being consistent with the observed checkerboard pattern. This relationship allows us to extract additional information about TAD organization than is possible from experiment alone [11]. It is carried out in two steps. (1) Since the contact probability is a function of both s and their epigenetic states, it is necessary to minimize the effect of the genomic separation in order to highlight the enrichment of contacts between loci of the same epigenetic state in the compartments. The correlation between the same type of loci is more transparent if the Spearman correlation matrix is used because it is based on the rank order (see above). (2) We treat the Spearman correlation matrix, $\mathbf{A}$, as an Adjacency matrix, where the vertices are the loci and the edge weight between the ith and j^{th} loci is the Spearman correlation coefficient, ρ_{ij}.

With these two steps, our clustering problem can be solved by finding the minimum cut vertex in a bipartite graph between the loci. This problem was solved by Dhillon using a spectral co-clustering algorithm [12] in a different context (clustering of documents and words). It is noteworthy that the underlying assumption of this method for our problem is that a pair of loci with positive Spearman correlation coefficient should be the same type. Similarly, a pair of loci with negative Spearman correlation coefficient should be distinct.

The Dhillon spectral biclustering algorithm is implemented as follows [12]:

1. Given the Spearman correlation map, $\mathbf{A}$, construct

$$\mathbf{A}_n = \mathbf{D}^{-1/2}\mathbf{A}\mathbf{D}^{-1/2}. \tag{F1}$$

2. Compute the left and right second singular vectors of $\mathbf{A}_n$, $\mathbf{u}_2$ and $\mathbf{v}_2$ and form the vector z_2 using,

$$\mathbf{z}_2 = \begin{bmatrix} \mathbf{D}^{-1/2}\mathbf{u}_2 \\ \mathbf{D}^{-1/2}\mathbf{v}_2 \end{bmatrix}. \tag{F2}$$

3. Perform the k-means algorithm on the 1-dimensional data z_2 to obtain the needed clustering.

The matrix $\mathbf{D}$ where $D_{ii} = \sum_j A_{ij}$ and $D_{ij} = 0$ for $i \neq j$ is the degree matrix of the graph. Note that by definition A and A_n are symmetric matrices. The left and right second singular vectors $\mathbf{u}_2$ and $\mathbf{v}_2$ would be the same. Thus, the simpler algorithm is to construct $\mathbf{z}_2 = \mathbf{D}^{-1/2}\mathbf{u}_2$ in step 2, and run step 3. The reason for using k-means clustering is that the values in $\mathbf{u}_2$ and $\mathbf{v}_2$ should have a bi-modal distribution, which is an approximation of the optimal two-valued partition vector [12]. The use of k-means algorithm allows us to find the two clusters corresponding to the bi-modal distribution.

Using the Dhillon's method, the Spearman correlation map $\mathbf{A}$ is bi-clustered into two clusters, with labeling vector $\mathbf{L}$, where $L_i = 1$ if the ith loci belongs to one cluster and $L_i = 0$ if the ith loci belongs to the other cluster. Note that swapping 0 and 1 in the labeling does not change the meaning.

The second step is to compare the cluster labeling between experiment and the prediction of the CCM. We denote the label assignment of the experimental data as $\mathbf{L}_{\text{exp}}$ and that extracted from the simulations as $\mathbf{L}_{\text{sim}}$. To measure the similarity between $\mathbf{L}_{\text{exp}}$ and $\mathbf{L}_{\text{sim}}$, we use the Adjusted Mutual Information score (AMI) measure. The Mutual Information score (MI) is,

$$\text{MI}(\mathbf{L}_{\text{exp}}, \mathbf{L}_{\text{sim}}) = \sum_{i=1}^{2}\sum_{j=1}^{2} P(i,j)\log\Big(\frac{P(i,j)}{P(i)P'(j)}\Big). \tag{F3}$$

where $P(i) = |L^i_{\text{exp}}|/N$ is the probability that a loci (monomer) picked at random from $\mathbf{L}_{\text{exp}}$ falls into type i, L^i_{exp} is the set of loci (monomers) of type i, and N is the total number of loci (monomers). Similarly, $P'(j) = |L^j_{\text{sim}}|/N$. In the above equation, $P(i,j) = |L^i_{\text{exp}} \cap L^j_{\text{sim}}|/N$ is the probability that a locus picked at random belongs to both set L^i_{exp} and L^j_{sim}. Since the expected

value of mutual information is non-zero, it is preferable to define the normalized AMI,

$$\text{AMI}(\mathbf{L}_{\text{exp}}, \mathbf{L}_{\text{sim}}) = \frac{\text{MI}(\mathbf{L}_{\text{exp}}, \mathbf{L}_{\text{sim}}) - E[\text{MI}(\mathbf{L}_{\text{exp}}, \mathbf{L}_{\text{sim}})]}{\max\{H(\mathbf{L}_{\text{exp}}), H(\mathbf{L}_{\text{sim}})\} - E[\text{MI}(\mathbf{L}_{\text{exp}}, \mathbf{L}_{\text{sim}})]} \tag{F4}$$

where $H(\mathbf{L}_{\text{exp}}) = -\sum_{i=1}^{2} P(i)\log\big(P(i)\big)$ and $H(\mathbf{L}_{\text{sim}}) = -\sum_{j=1}^{2} P'(j)\log\big(P'(j)\big)$. In the above equation, $E[\text{MI}(\mathbf{L}_{\text{exp}}, \mathbf{L}_{\text{sim}})]$ is the expected value of the mutual information, which can be calculated using the following equation [13],

$$\sum_{i=1}^{2}\sum_{j=1}^{2}\sum_{n_{ij}=(a_i+b_j-N)^+}^{\min(a_i,b_j)} \frac{n_{ij}}{N}\log\left(\frac{Nn_{ij}}{a_ib_j}\right)\frac{a_i!b_j!(N-a_i)!(N-b_j)!}{N!n_{ij}!(a_i-n_{ij})!(b_j-n_{ij})!(N-a_i-b_j+n_{ij})!} \tag{F5}$$

where $a_i = |L^i_{\text{exp}}|$ and $b_j = |L^j_{\text{sim}}|$, and $(a_i + b_j - N)^+$ denotes $\max(1, a_i + b_j - N)$.

Supplementary Fig.9 compare the AMI between experimental data and results from simulations. The AMI scores for the CCM model are significantly higher than those for the homopolymer model. Thus the long-range compartment pattern can only be obtained using the minimal CCM or other copolymer model.

Supplementary Note 9: Ward Linkage Matrix

The method described in the previous section allows for a quantitative comparison of simulated and measured contact maps. However, it cannot be used as a measure to compare 3D structures (spatial patterns obtained in super-resolution experiments, for example) of chromosomes. In order to achieve this goal, we first relate the information contained in the contact maps to spatial distances. As shown in Fig.3b in the main text, the contact probability is inversely proportional to a power of the spatial distance, $P(s) \propto R(s)^{-4.1}$ which provides a way to convert a Hi-C contact matrix to the spatial distance matrix, differing from the physical spatial distance matrix by only a constant prefactor. We then compare the "pseudo" spatial distance matrix with our simulated spatial distance matrix. Needless to say that in simulations R_{ij} can be directly computed.

Matrix norm is often used to measure the distance between two matrices. However, it has severe drawbacks in the context of chromosome organization for two reasons. First, the

element-wise differences cannot capture the similarities of higher order structure embedded in the matrix. Second, it suffers from "curse of dimensionality" [14], i.e. there is little difference in the distances between different pairs of matrices, which makes it impossible to differentiate between the experimentally inferred spatial distance matrix and the matrices obtained in the simulations with different parameters. To overcome these difficulties, we adopted the method described recently [15], which suggests treating the original matrix as a graph where the matrix element is a measure of the distance (which is naturally satisfied in our context), and transform it to a cophenetic matrix. In the process, the topological structure of the information embedded in the matrix is preserved. By adopting this method, we can compare the simulated structures of the folded chromosomes with that inferred from Hi-C data.

We converted the Hi-C contact matrix to a "pseudo" spatial distance matrix $\mathbf{R}_{\text{exp}}$, using the relation $R_{ij} = P_{ij}^{-1/4.1}$ ($|i-j| \propto s$). We constructed the Ward Linkage Matrix (WLM), $\mathbf{W}$, from $\mathbf{R}_{\text{exp}}$ and the simulated spatial distance matrices $\mathbf{R}_{\text{sim}}$. The algorithm to construct a WLM is the following. First, start with each locus in a cluster of its own. Second, find the pair of clusters with the smallest Ward distance (see below) and merge them. Third, repeat the second step until there is only one cluster. Finally, the WLM is constructed as follows. Suppose i and j belong to two disjoint clusters S and T and are joined by a *direct* parent cluster. The entry of the WLM, w_{ij}, is the Ward distance between clusters S and T, given by,

$$d(S,T) = \left(\sum_{i \in S \cup T} ||\mathbf{x}_i - \mathbf{c}_{S\cup T}||^2 - \sum_{i\in S} ||\mathbf{x}_i - \mathbf{c}_S||^2 - \sum_{i \in T} ||\mathbf{x}_i - \mathbf{c}_T||^2 \right)^{1/2} = \left(\frac{n_S n_T}{n_S + n_T} ||\mathbf{c}_S - \mathbf{c}_T||^2 \right)^{1/2} \tag{G1}$$

where $\mathbf{c}_S$ and $\mathbf{c}_T$ are the centers of S and T, respectively; n_S (n_T) is the number of monomers in S (T), and $\mathbf{x}_i$ is the position of point i. The initial clustering occurs between singleton clusters (cluster on its own). The distance between two singleton clusters, i and j, is,

$$d(i,j) = ||\mathbf{x}_i - \mathbf{x}_j|| = (\mathbf{x}_i - \mathbf{x}_j)^{1/2} \tag{G2}$$

which in our case is simply R_{ij}, the spatial distance between the ith and j^{th} loci.

In practice, we used the Lance-Williams recursive algorithms [16] to compute the Ward

distance. Suppose we have three clusters C_i, C_j and C_k, and the Ward distances between them, $d(C_i, C_k)$, $d(C_j, C_k)$ and $d(C_i, C_j)$, are known. The Ward distance between the union of clusters i and j, $C_i \cup C_j$ and C_k, is obtained using the recursive equation,

$$d(C_i \cup C_j, C_k) = \left(\frac{n_i + n_k}{n_i + n_j + n_k} d^2(C_i, C_k) + \frac{n_j + n_k}{n_i + n_j + n_k} d^2(C_j, C_k) - \frac{n_k + n_k}{n_i + n_j + n_k} d^2(C_i, C_j) \right)^{1/2} \tag{G3}$$

where C_i, C_j and C_k are disjoint clusters with sizes n_i, n_j and n_k.

Supplementary Note 10: Shape of TADs

We use shape parameters, to investigate the shape of the 32 TADs in Chr 5 (Table I) formed by loop anchors. We calculated three metrics to quantify the shapes, radius of gyration R_g, relative shape anisotropy, κ^2 and shape parameter, S. The value of R_g^2 is,

$$R_g^2 = \lambda_1 + \lambda_2 + \lambda_3, \tag{H1}$$

where λ_i are the eigenvalues of the gyration tensor; κ^2 is defined as,

$$\kappa^2 = \frac{3}{2} \frac{\lambda_1^2 + \lambda_2^2 + \lambda_3^2}{(\lambda_1 + \lambda_2 + \lambda_3)^2} - \frac{1}{2}. \tag{H2}$$

The shape parameter, S, is,

$$S = 27 \prod_{i=1,2,3} (\lambda_i - \bar{\lambda})/\bar{\lambda} \tag{H3}$$

where $\bar{\lambda} = (\lambda_1 + \lambda_2 + \lambda_3)/3$. The bounds for κ^2 is $0 \leq \kappa^2 \leq 1$, where 0 is for a highly symmetric conformation and 1 corresponds to a rod, S satisfies $-1/4 \leq S \leq 2$. If $-0.25 < S < 0$, then the shape is predominantly oblate and is prolate for $0 < S < 2$ [17, 18]. The results in the Supplementary Fig.10 (Left panel) show R_g^2, κ^2 and S measurements for the CCM for $\epsilon = 2.4 k_{\mathrm{B}}T$. The top figure shows that R_g increases as the size of the TAD increases. Both the middle and bottom figures show that small TADs deviate from spherical shape (large value of κ^2 and S) but adopt a more spherical shape as the size of TAD increases.

We also calculated the dispersion in the three measurements for each TAD among trajectories (right column in Supplementary Fig.10). For instance, dispersion of the radius of

gyration is defined as $\sigma_{R_g^2}/\mu_{R_g^2}$, where $\sigma_{R_g^2}$ and $\mu_{R_g^2}$ are the standard deviation and mean value of $\overline{R_g^2}$ over trajectories and the bar denotes the time average. The histograms of $\overline{R_g^2}$, $\overline{\kappa^2}$ and $\overline{S}$ normalized by their mean values are shown in Supplementary Fig.11.

Supplementary Note 11: Dispersion of spatial distance $R(s)$

The differences in the structural properties between various cells can be glanced from the variance among independent trajectories. To quantify the heterogeneity of the structure of the chromosome, we computed both the coefficient of variation, $\delta R(s)$, and variance of the spatial distance $R(s)$, $\sigma^2_{R(s)}$. Here, $\delta R(s) = (\langle R(s)^2 - \langle R(s)\rangle^2)^{1/2}/\langle R(s)\rangle = \sigma_{R(s)}/\mu_{R(s)}$, where $\langle\cdot\rangle$ is average over independent trajectories, and $R(s) = \overline{\sum_{i<j}^{N} \frac{(\mathbf{r}_i-\mathbf{r}_j)^2\delta(s-|i-j|)}{N-s}}^{1/2}$ with the bar denoting time average along single trajectory. The variance of $R(s)$ is $\sigma^2_{R(s)} = \langle R^2(s)\rangle - \langle R(s)\rangle^2$. Supplementary Figs.12a, b show the results for $\epsilon = 1.0k_{\mathrm{B}}T$ and $\epsilon = 2.4k_{\mathrm{B}}T$. Coefficient of variation and variance of $R(s)$ are also computed using the experimental data [11] (Supplementary Figs.12c, d). $\sigma^2_{R(s)}$ increases with s for $s < 10^6$ bps for both $\epsilon = 1.0k_{\mathrm{B}}T$ and $\epsilon = 2.4k_{\mathrm{B}}T$. It reaches plateau for $\epsilon = 1.0k_{\mathrm{B}}T$ for $s > 10^6$ bps whereas it continues to increase for $\epsilon = 2.4k_{\mathrm{B}}T$. Experimental data (Supplementary Fig.12d) show that $\sigma^2_{R(s)}$ consistently increases for s up to several 10^7 bps. More interestingly, curves for $\delta R(s)$ for the liquid-like system and glassy-like system have very different shapes. In the glassy state, $\delta R(s)$ reaches a peak $\sim 10^5$bps and decrease for $s > 10^5$bps, whereas $\delta R(s)$ in the mobile state reaches a plateau at $s \sim 10^6$ bps. Surprisingly, the decrease in $\delta R(s)$ for large s can be seen in the experimental data (Supplementary Fig.12c), further supporting our conclusion that chromosomes dynamics is glassy.

Supplementary Note 12: Chromsome 10

In order to check the transferability of the CCM, we obtained the contact maps of Chr 10 with the same set of parameters (Supplementary Table II) used for simulating Chr5. The locations of loop anchors used in Chr 10 simulation are summarized in Supplementary Table III. The

chromatin segments selected is from 70 to 82 Mbps. The types of monomers (chromatin loci) are determined using the Broad ChromHMM track, as described earlier. The numbers of active and repressive monomers are 3980 and 6020, respectively. About 80% of compartments inferred from experiment Hi-C contact map are correctly predicted by the CCM.

For precise comparison between the prediction of the CCM and experiment, we computed the Ward Linkage Matrices. Supplementary Fig.16 shows the WLM inferred from experiment and computed directly from simulations. Just as for Chr 5, visual inspection suggests that $\epsilon = 2.4 k_{\mathrm{B}}T$ and $\epsilon = 2.7 k_{\mathrm{B}}T$ agrees best with experiments. To quantitatively compare the WLMs, we compute the Pearson correlation coefficient between experimental WLM and simulated WLMs. The values of Pearson correlation coefficients are 0.58, 0.75, 0.92 and 0.92 for $\epsilon = (1.0, 2.0, 2.4, 2.7)k_{\mathrm{B}}T$, respectively, suggesting that $\epsilon = 2.4 k_{\mathrm{B}}T$ and $\epsilon = 2.7 k_{\mathrm{B}}T$ give excellent agreement with experiments. This is consistent with our detailed study on Chr 5. These results show that the minimal CCM is sufficient to capture the features of the folded chromosomes. Applications of the CCM to other chromosomes are planned.

Supplementary Note 13: $F_{\mathrm{s}}(k, t)$ and $\chi_4(k, t)$ for two types of chromatin loci

Supplementary Fig.18a shows $F_{\mathrm{s}}(k, t)$ computed for active and repressive loci for two different values of ϵ evaluated at $k = 2\pi/r_s$, where r_s is the position of the first peak of radial distribution function $g(r)$ (Fig.2 in the main text). For $\epsilon = 1.0 k_{\mathrm{B}}T$, both active and repressive loci exhibit similar relaxation behavior. However, in the glassy state, repressive loci have longer relaxation times, although the decay of both $F_s(k, t)$ for active and repressive loci can be fit using stretched exponential functions. Supplementary Fig.18b shows the dynamical fourth order susceptibility $\chi_4(k, t)$. In the glassy state, active loci exhibit a similar peak shifted to slightly lower t compared to repressive loci and the position of the peak ($t \sim 0.1$s) is substantially smaller than it for the repressive loci ($t \sim 1$s). We surmise that the structure of active compartments relaxes faster than repressive compartments, whereas repressive compartments are more “glassy” than active compartments.

We also report in Supplementary Figs.18c, d the intermediate scattering function $F_s(k,t)$ and $\chi_4(k,t)$ for different values of k. Values of k, measured in the unit of σ^{-1}, are taken to be 5.738, 3.281, 2.256 and 1.717, corresponding to the first, second, third and fourth peak of radial distribution function $g(r)$. Not unexpectedly, the relaxation times decrease as the length scale decreases. Interestingly, at all scale probed in Supplementary Fig.18 it appears that the overall decay is slow. This implies that at all relevant (from a structural organization perspective) genomic distances, chromosomes exhibit glass-like characteristics (Supplementary Figs.18c, d)

Supplementary Note 14: Details of scaling between $P(s)$ and $R(s)$

The analysis of the relationship between $P(s)$ and $R(s)$ ($P(s) \propto R(s)^{-\lambda}$) reveals that the scaling exponent λ is s-dependent. Supplementary Fig.19 shows $1/P(s)$ as a function of $R(s)$. $1/P(s) \propto R(s)^{\lambda}$ with $\lambda \approx 2$ for $R(s) \lessapprox 0.5\mu$m and $\lambda \approx 6$ for $R(s) \gtrapprox 0.7\mu$m. Fitting over the whole range of $R(s)$ gives $\lambda \approx 4.1$, which is observed in the experiment[11]. Interestingly, the Fig.1G in [11] suggests that the value of λ appears to be smaller than 4.1 on short length scale and greater than 4.1 for large $R(s)$, consistent with what is shown in Supplementary Fig.19.

The relation between $P(s)$ and $R(s)$ is only meaningful in an average sense. Given the value of $P(s)$, the structure derived from relation $P(s)^{-1/\lambda}$ can only be interpreted as an "ensemble/population averaged" structure. This is vividly demonstrated in Fig.5 in the main text. It shows that although each individual chromosome structure varies greatly the ensemble averaged simulated structure of chromosome agrees well with experiments in the sense that the Hi-C contact map and the average distance map from FISH experimentally emerge from the CCM.

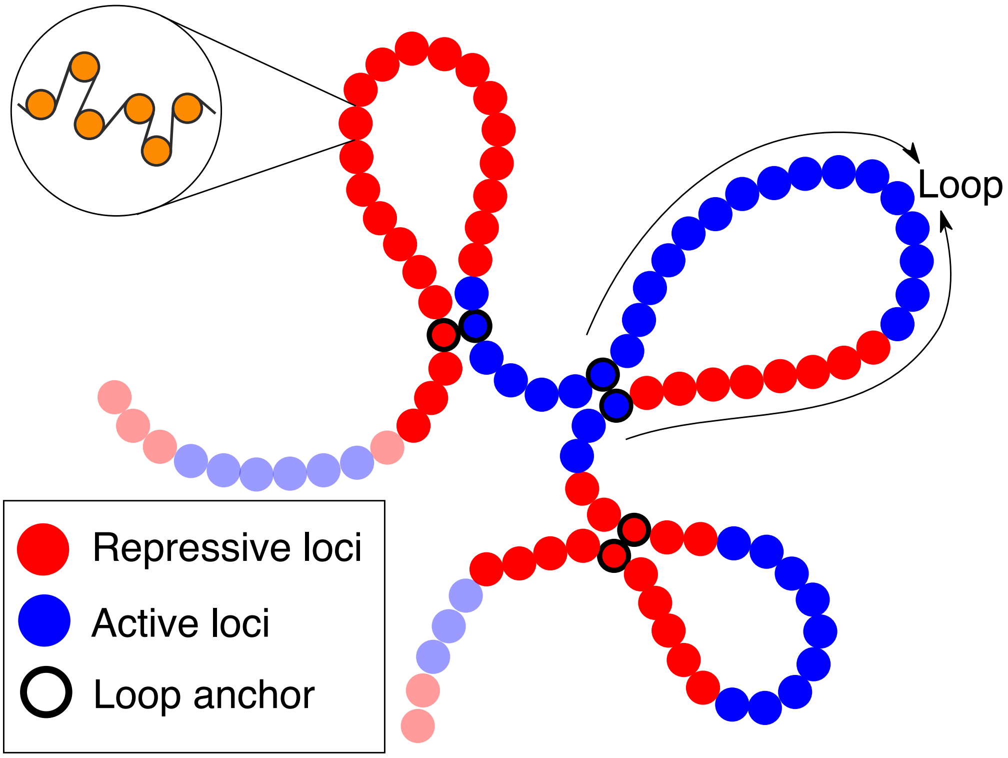

Supplementary Figure 1: The sketch of the Chromosome Copolymer Model (CCM). Each bead represents $1,200$ basepairs (representing roughly six nucleosomes (orange circles) connected by linker DNAs). Red (Blue) corresponds to repressive (active) chromatin. The three pairs of loop anchors (in this cartoon) are marked by beads with black boundaries. A crucial aspect of the model, based on the experimental observation [1] is that the loops are consecutive and do not overlap with each other. The CCM accounts for two epigenetic states and the locations of the loop anchors. These two criteria are sufficient to reproduce all the subtle structural features noted in the Hi-C and super-resolution experiment.

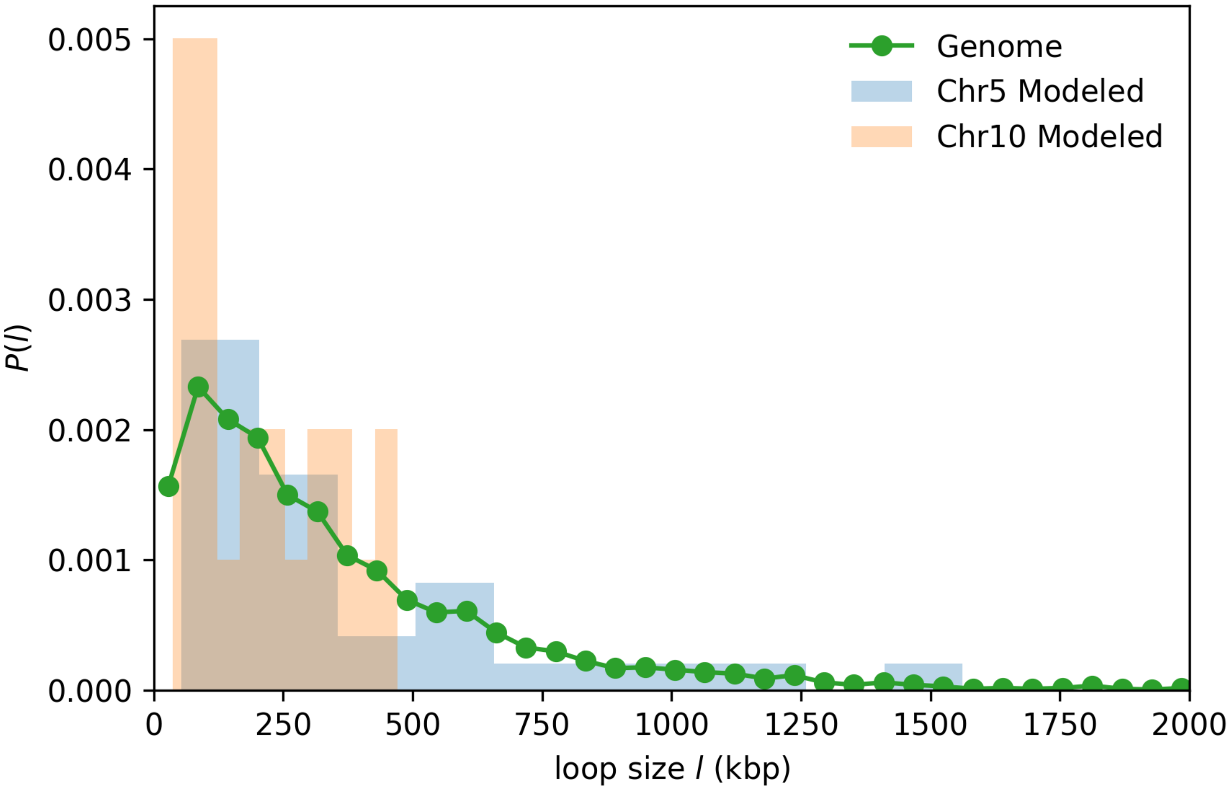

Supplementary Figure 2: The distribution of loop size l, $P(l)$, where l is the genomic separation between the anchors. The blue bar (orange bar) is the $P(l)$ for the selected range of Human Chromosome 5 (Chromosome 10) modeled in this work. The green curve is the $P(l)$ for the whole genome of the 23 chromosomes [1].

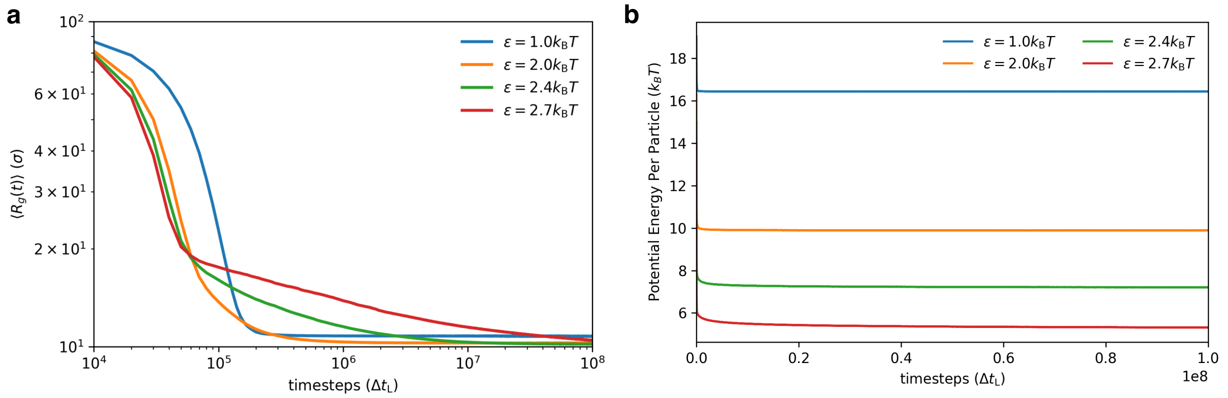

Supplementary Figure 3: Preparation of the initial conformations. (**a**) The ensemble average radius of gyration $\langle R_{\rm g}(t)\rangle$ as function of time step t after the attractive interactions are turned on. $\langle R_{\rm g}(t)\rangle = (1/{\rm M})\sum_{i=1}^{\rm M} R_{\rm g}^{(i)}(t)$ where i is the $i^{\rm th}$ trajectory, and M is total number of independent trajectories. $R_{\rm g}^{(i)}(t)$ is the radius of gyration of trajectory i at time t. In our simulations, ${\rm M} = 90$. (**b**) The average potential energy per bead as a function of the number of time steps t after the attractive interactions are turned on. The average is over the 90 independent trajectories. The plateau in (**a**) and (**b**) suggest that the polymer conformations are well sampled.

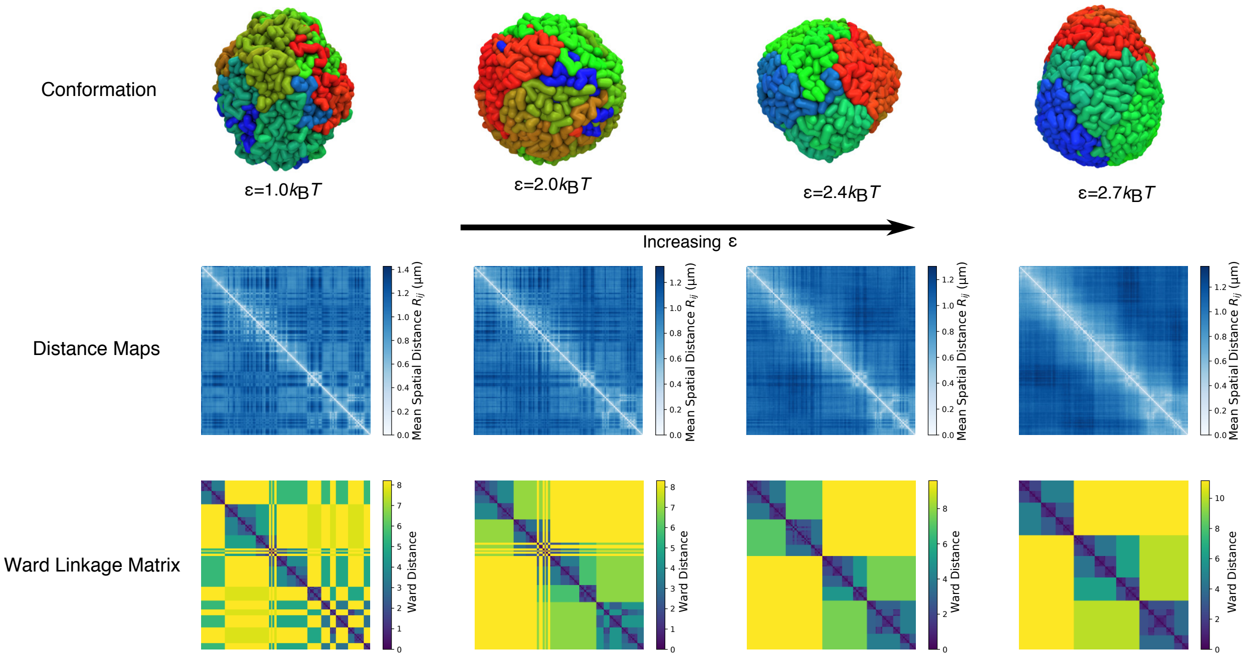

Supplementary Figure 4: The top panel shows typical structures of the simulated folded Chr5 for $\epsilon = (1.0, 2.0, 2.4, 2.7)k_BT$ from left to right. The color indicates the index of the locus, from the $5'$ to $3'$-end. The spatial distance map and the corresponding Ward Linkage Matrices (WLMs) are shown in the middle and bottom panels, respectively.

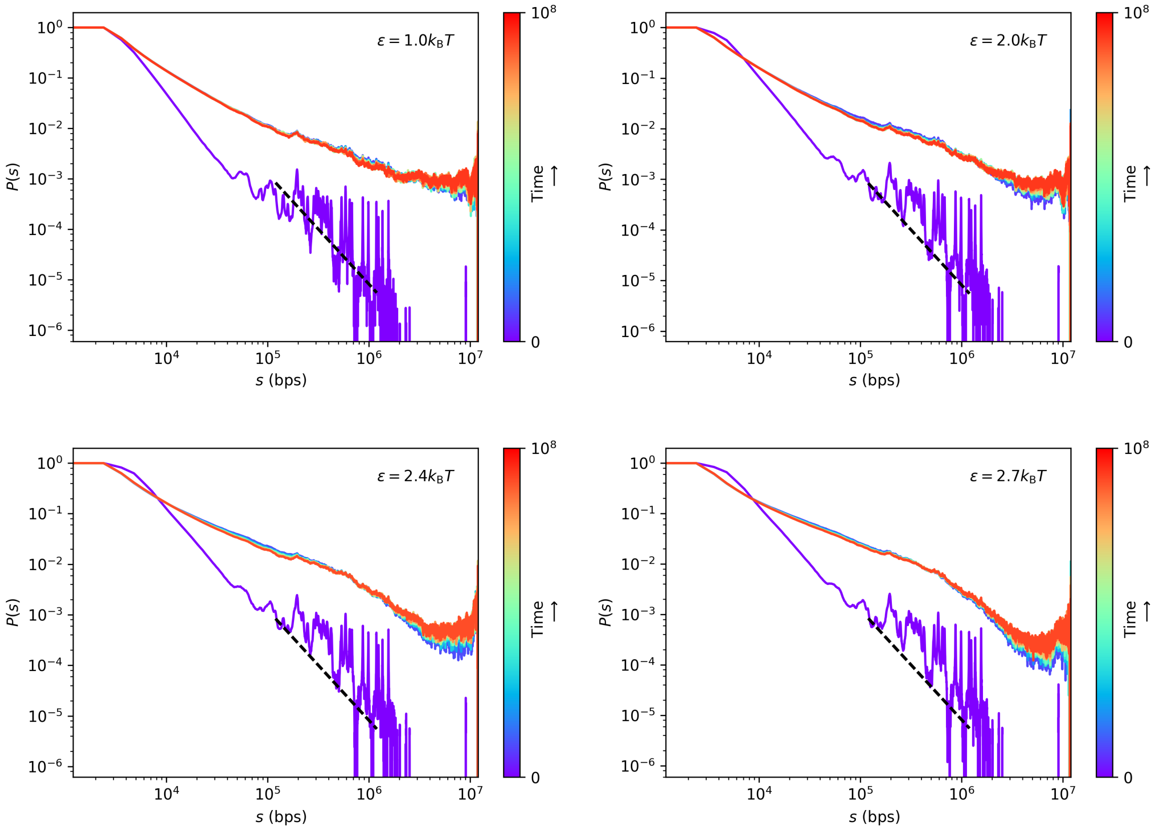

Supplementary Figure 5: The time evolution of the contact probability $P(s)$ as a function of s during Chr 5 folding calculated using Langevin Dynamics simulations. The four graphs are for different ϵ values (Supplementary Table II). The color bar on the right marks the value of time t in the unit of $\Delta t_{\rm L}$ (see the section "Simulation details"). At $t = 0$, the chromosome is swollen (purple curve). The slope of the dashed black line is $\Theta_3 = 2.181$, which is the value of Des Cloizeaux [19] prediction for Θ_3 derived for long homopolymers in a good solvent. The Θ_3 exponent describes the probability $P(s)$ of forming a loop of size s between two interior points and is given by $P(s) \sim s^{-\Theta_3}$. After the initial condensation of the chromosome, the copolymer adopts compact conformation with a shallower s-dependent of $P(s)$ at all ϵ values. The change in $P(s)$ as a function of time t is small, suggesting that the time evolution of $P(s)$ is sluggish

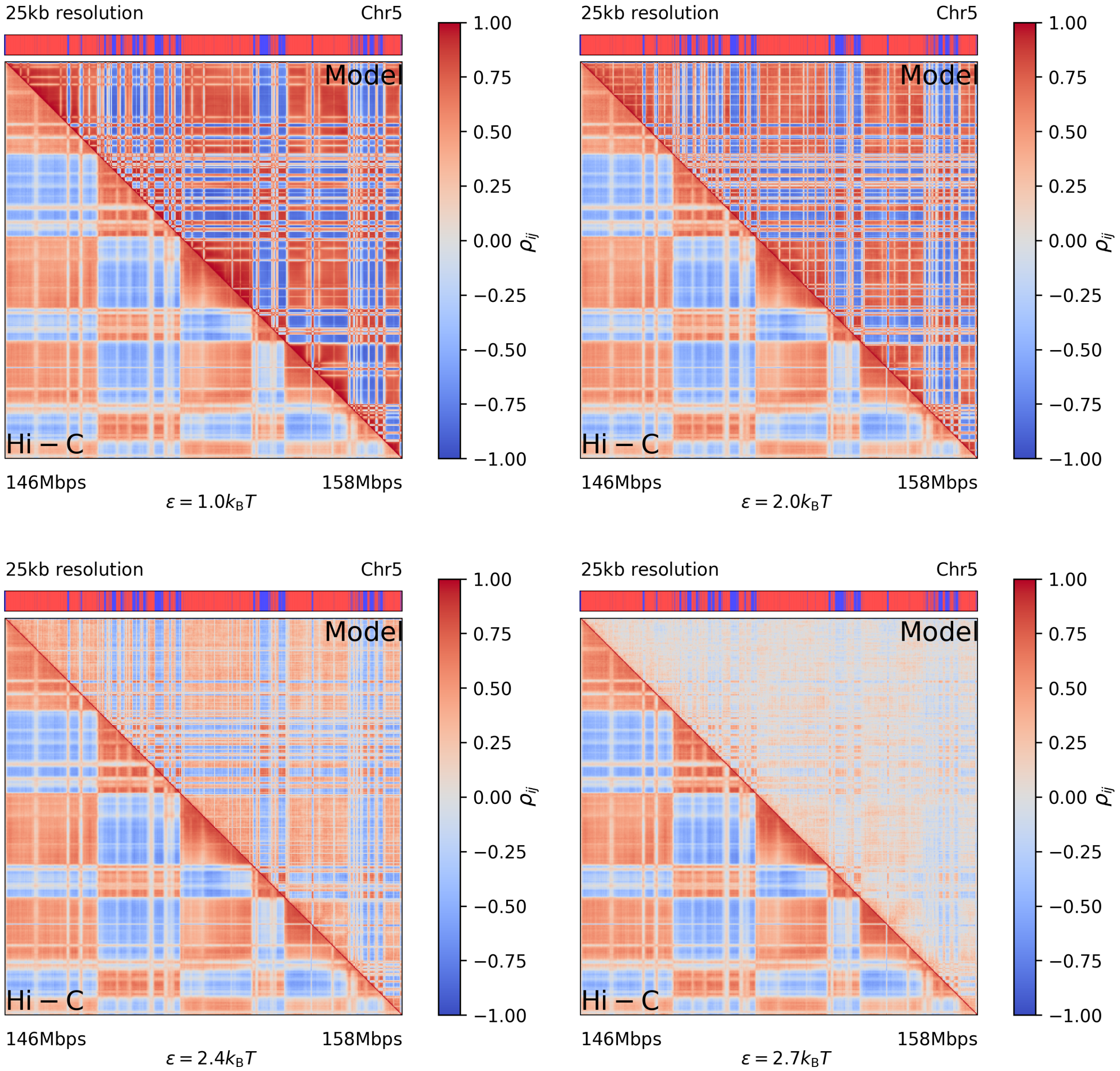

Supplementary Figure 6: Spearman correlation map computed for $\epsilon = (1.0, 2.0, 2.4, 2.7)\mathrm{k_B}T$. For each figure, left lower triangle is the Spearman correlation map computed from the Hi-C data, and the upper right triangle is the simulated map. The color bar shows the value of the Spearman correlation coefficient with the value of 1 (-1) indicating perfect correlation anti-correlation; 0 implies no correlation. When the copolymer goes from displaying liquid-like behavior ($\epsilon < 2.4\mathrm{k_B}T$) to exhibiting glassy dynamics ($\epsilon > 2.4\mathrm{k_B}T$), the distinction between anti-correlation (blue) and correlation (red) becomes less transparent. Note that the agreement between simulation and experiment is best for $\epsilon = 2.4\mathrm{k_B}T$.

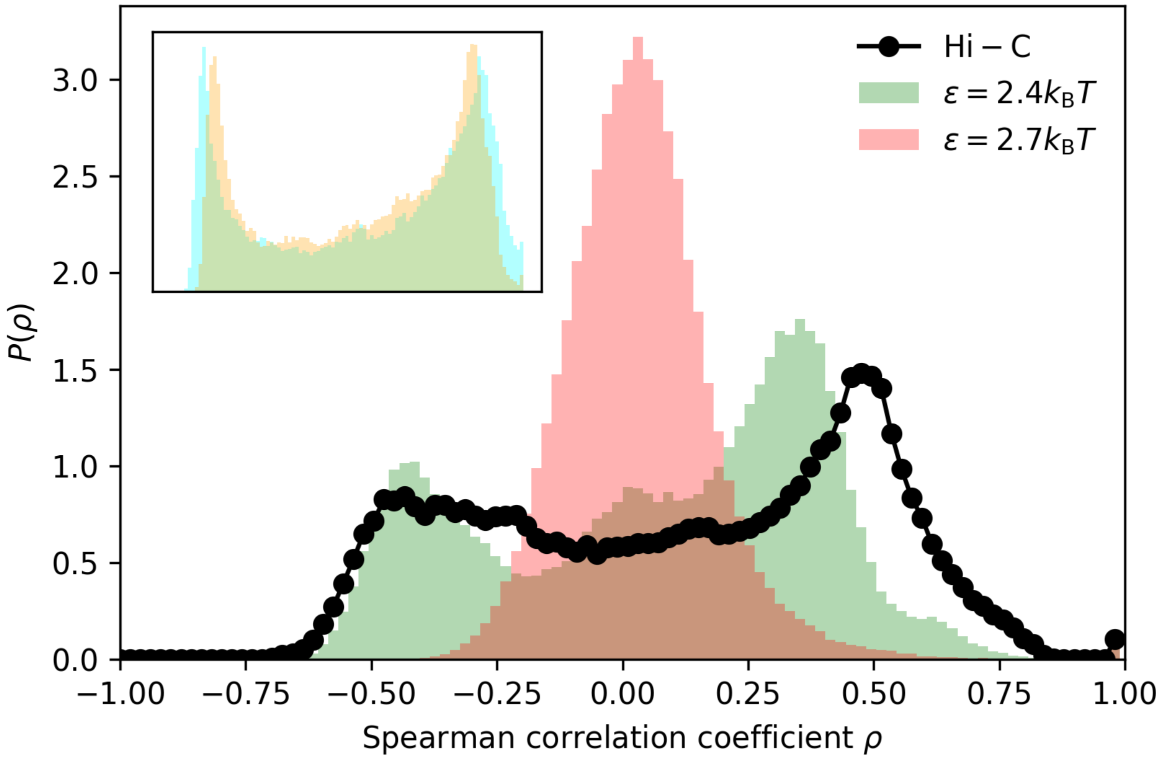

Supplementary Figure 7: Comparison of the histograms of the Spearman correlation coefficient, ρ, from simulations and experiment. We plot the distribution of ρ_{ij} for every pair of (i, j). The black line is from the Hi-C experiment. The bimodal shape of the distribution is a result of two different compartment patterns in the Hi-C map. The inset shows the distribution for $\epsilon = 1.0k_BT$ (cyan) and $\epsilon = 2.0k_BT$ (orange). As the dynamics becomes increasingly glassy, the extent of bimodality becomes weaker and exhibits only one peak for $\epsilon = 2.7k_BT$. The closest agreement between simulations and the experiment data occurs when $\epsilon = 2.4k_BT$, thus justifying this value in simulating Chr 5 and 10.

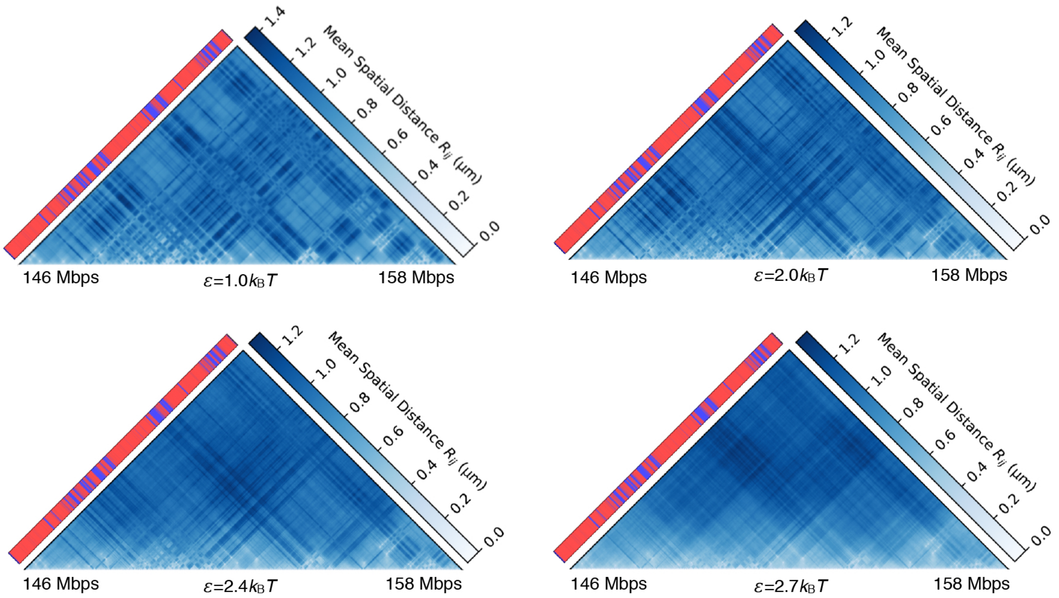

Supplementary Figure 8: Spatial distance map for $\epsilon = (1.0, 2.0, 2.4, 2.7)k_\mathrm{B}T$. Each element in the distance map is $R_{ij} = \langle|\mathbf{r}_i - \mathbf{r}_j|\rangle$ where the bracket $\langle\cdot\rangle$ is the average over both ensembles (trajectories) and time. The color bar gives the value of R_{ij} in the unit of μm.

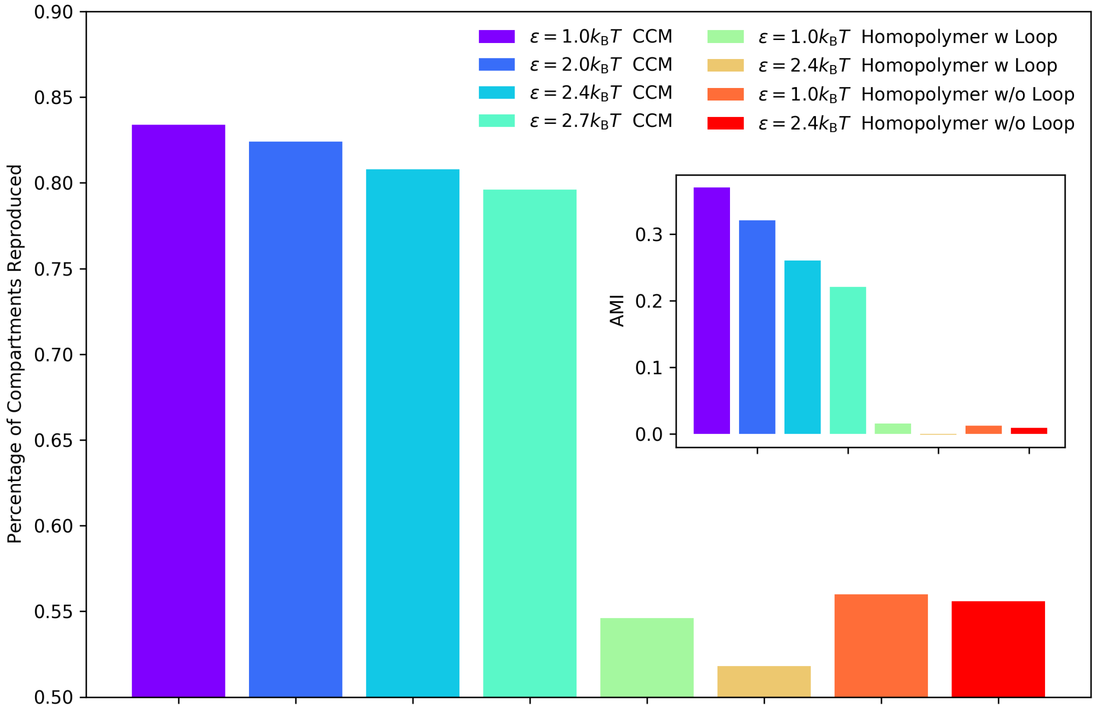

Supplementary Figure 9: Percentage of correctly predicted compartments based Adjusted Mutual Information score (AMI) for the CCM and homopolymer models. CCM correctly reproduces $\approx 83\%, 82\%, 81\%, 80\%$ of the compartments found in experiments for $\epsilon = (1.0, 2.0, 2.4, 2.7)k_\mathrm{B}T$, respectively. The values for the homopolymer are very low, which implies that it cannot capture the structures of the chromosomes. The inset shows the AMI score for different cases. Note that AMI score is more sensitive compared to the percentage of compartments reproduced.

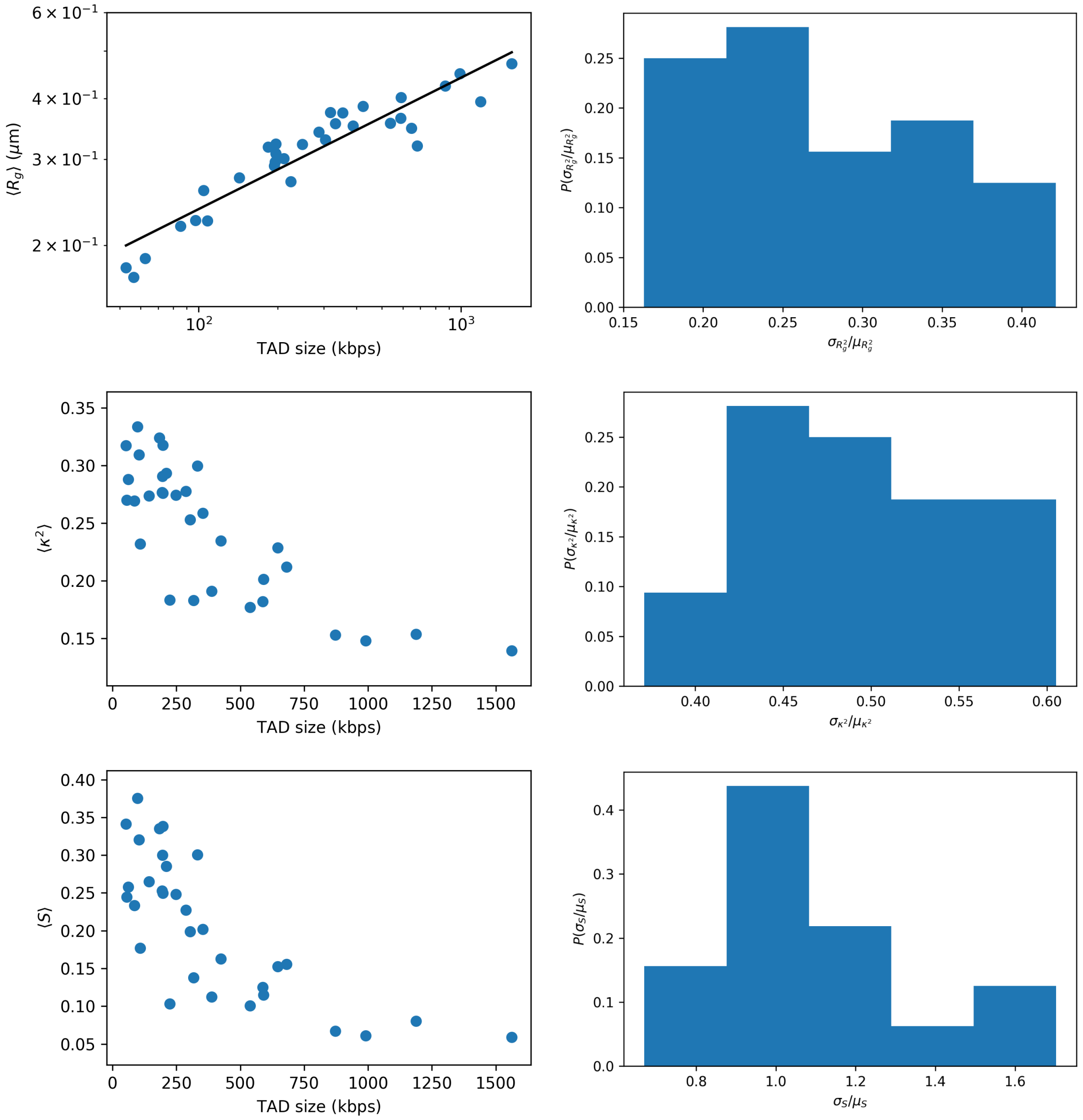

Supplementary Figure 10: **(Left panel)** $\langle R_g^2 \rangle$(top) (Eq.H1), $\langle \kappa^2 \rangle$(middle) (eq.H2) and $\langle S \rangle$(bottom) (eq.H3) for each TAD, where $\langle \cdot \rangle$ denotes both ensemble and time average. The black line in the top figure is the fit to the data, $\langle R(g) \rangle \sim l^{0.27}$, where l is the TAD size. **(Right panel)** Distribution $P(\sigma_{R_g^2}/\mu_{R_g^2})$(top), $P(\sigma_{\kappa^2}/\mu_{\kappa^2})$(middle) and $P(\sigma_S/\mu_S)$(bottom) over all TADs. $\sigma_{R_g^2} = \left[\langle \overline{R_g^2}^2 \rangle - \langle \overline{R_g^2} \rangle^2\right]^{1/2}$, $\sigma_{\kappa^2} = \left[\langle \overline{\kappa^2}^2 \rangle - \langle \overline{\kappa^2} \rangle^2\right]^{1/2}$ and $\sigma_{S^2} = \left[\langle \overline{S^2}^2 \rangle - \langle \overline{S^2} \rangle^2\right]^{1/2}$ where $\bar{\cdot}$ denotes time average over single trajectory and $\langle \cdot \rangle$ denotes ensemble average over different trajectories.

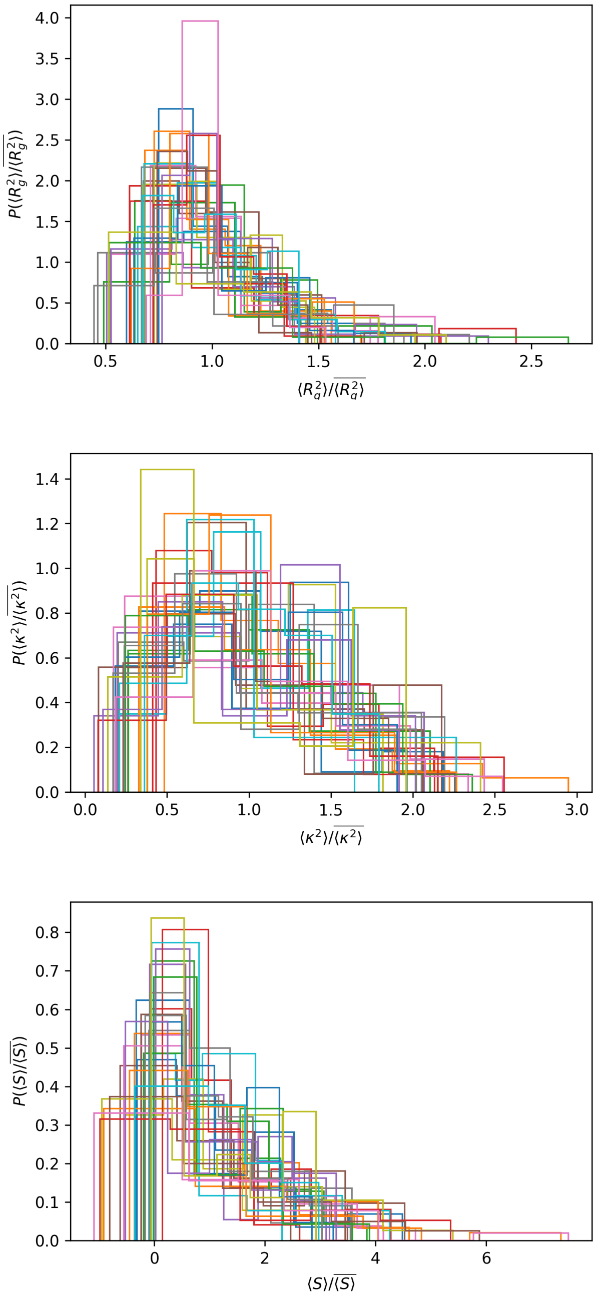

Supplementary Figure 11: The distribution of $\overline{R_g^2}/\langle\overline{R_g^2}\rangle$ for the thirty-two TADs in Chr5 where $\overline{R_g^2}$ is the time average value of the squared radius of gyration of single trajectory and $\langle\overline{R_g^2}\rangle$ is its mean value averaged over all independent trajectories. TADs are represented by different colors. Distribution of $\overline{\kappa^2}/\langle\overline{\kappa^2}\rangle$ and $\overline{S^2}/\langle\overline{S^2}\rangle$ are shown in the middle and bottom panels, respectively.

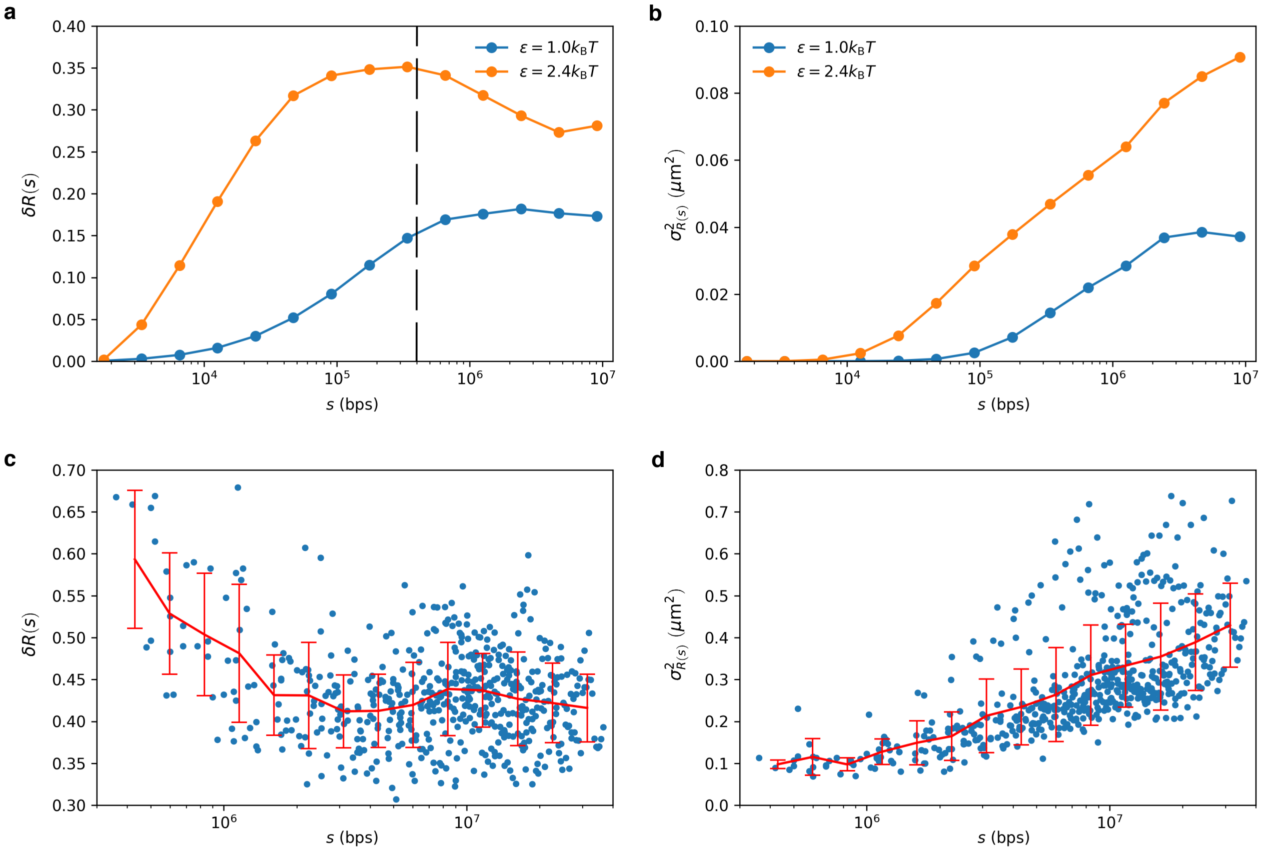

Supplementary Figure 12: Dependence of the coefficient of variation of $R(s)$ (**a**) and variance of $R(s)$ (**b**) averaged over the 90 trajectories on s. The value of s beyond the vertical dashed line is in the range probed in the experiment [11]. The experimental counterpart is computed from FISH data (Table S4 and Fig.S3 in Ref. [11]). The blue dots in (**c**) and (**d**) are computed from the distribution of spatial distance for pairs of TADs over the 120 individual cells reported in Ref. [11]. The s scale in the experiment covers $s \gtrapprox 4 \cdot 10^5$bps up to $\approx 3 \cdot 10^7$bps whereas in simulation s varies from 10^3bps to 10^7bps. For $s \gtrapprox 4 \cdot 10^5$bps the result for simulations and experiment are qualitatively similar. The red curve is the window average of the scatter in the data shown with error bars.

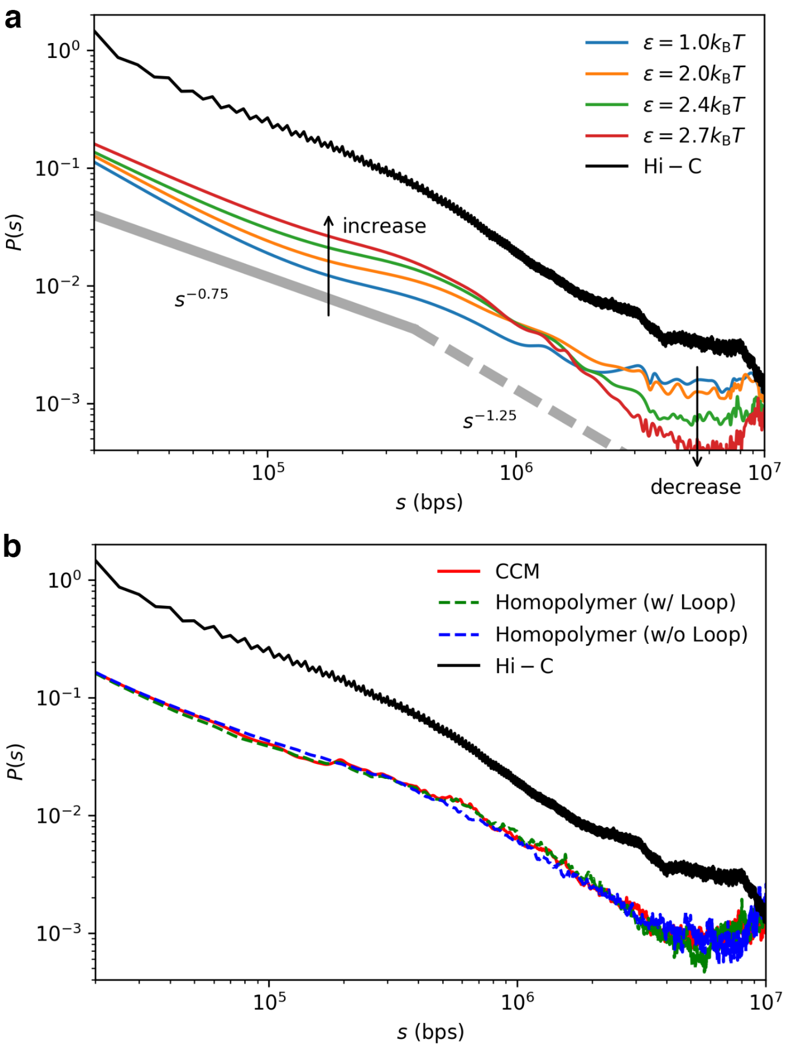

Supplementary Figure 13: **(a)** Contact probability $P(s)$ as a function of s for $\epsilon = (1.0, 2.0, 2.4, 2.7)k_{\mathrm{B}}T$. As ϵ increases, the local short-range contact probability ($\lessapprox 3 \cdot 10^5$ bps) increases by several folds, whereas the probability of non-local long-range contact ($\gtrapprox 3 \cdot 10^6$ bps) decreases. Black line is the Hi-C experimental result [1], which is displaced for a clear comparison. Solid and dashed grey lines demarcate the two scaling regimes in $P(s)$. **(b)**. $P(s)$ for the CCM (blue) and homopolymer with (green dashed) or without (red dashed) loop constraints. For the homopolymer, $\epsilon_{AA} = \epsilon_{BB} = \epsilon_{AB} = 2.4k_{\mathrm{B}}T$. The minor difference between the CCM, homopolymer model and Hi-C experiment suggests that $P(s)$ is too simple a measure for discriminating between different chromosome structural models.

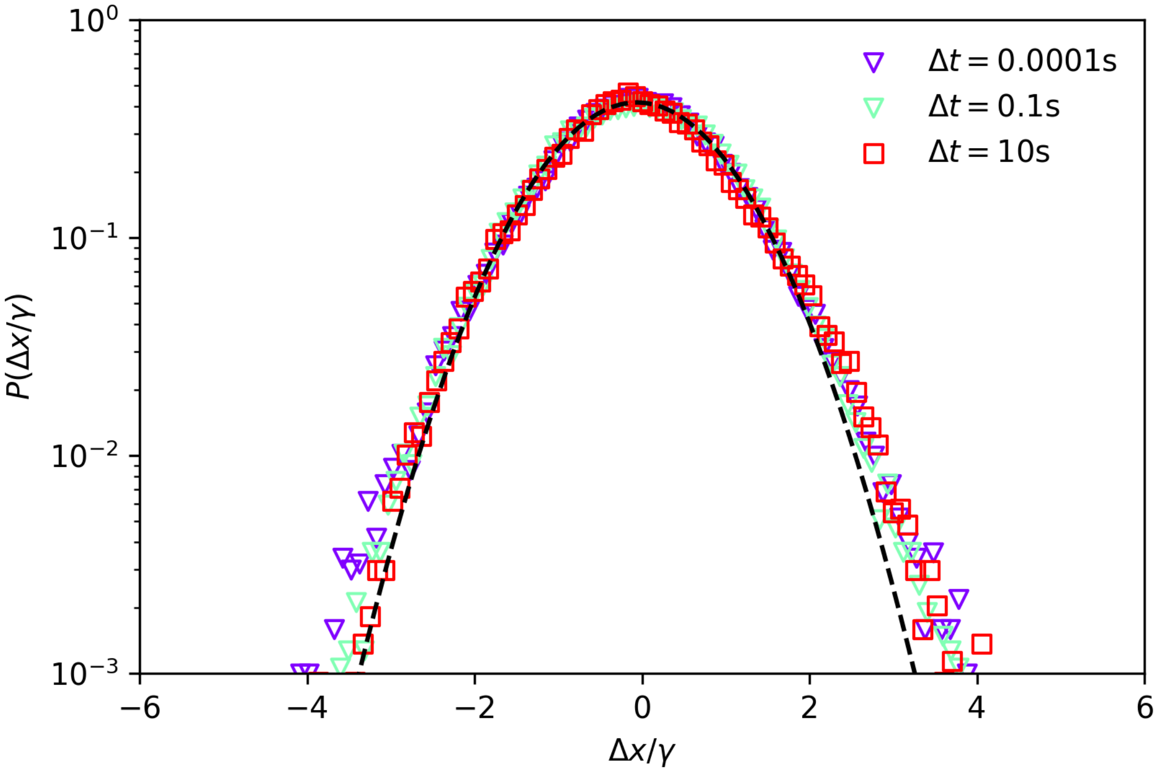

Supplementary Figure 14: The normalized Van Hove function $P(\Delta x/\gamma)$ for $\epsilon = 1.0k_{\mathrm{B}}T$ at various lag times Δt. γ is the standard deviation of Δx. The distributions ($P(\Delta x/\gamma)$) for different lag times collapse onto a master curve. Black dashed line is the Gaussian fit with mean -0.06 and standard deviation 0.95. The Gaussian behavior is in stark contrast with the dramatically non-gaussian behavior in the Chr 5 with $\epsilon = 2.4k_{\mathrm{B}}T$ (Fig.7d in the main text) - a value needed to reproduce the experimentally inferred structural features.

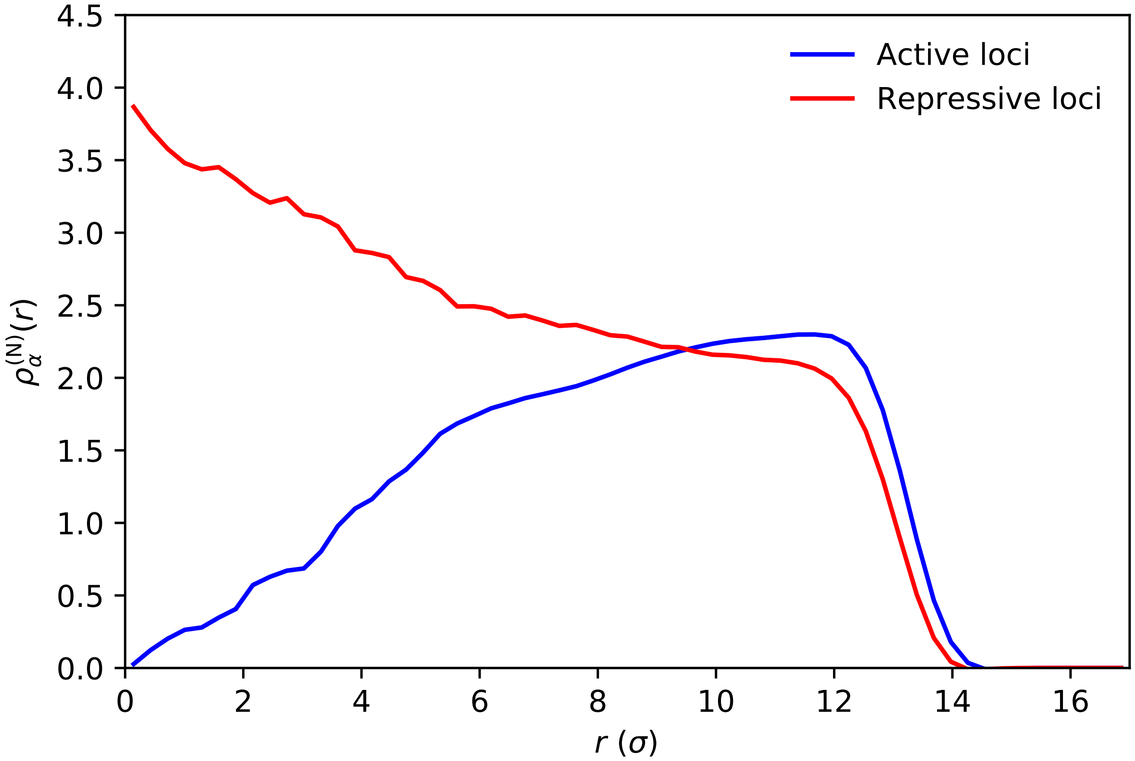

Supplementary Figure 15: The normalized radial density for Chr 10, $\rho_\alpha^{(\mathrm{N})}(r) = \langle N_\alpha(r)\rangle/(4\pi r^2 N_\alpha)$, where N_α is the number of loci of given type α found in the spherical shell between r and $r+\Delta r$, N_α is the total number of loci of that type. The bracket $\langle\cdot\rangle$ is the ensemble average.

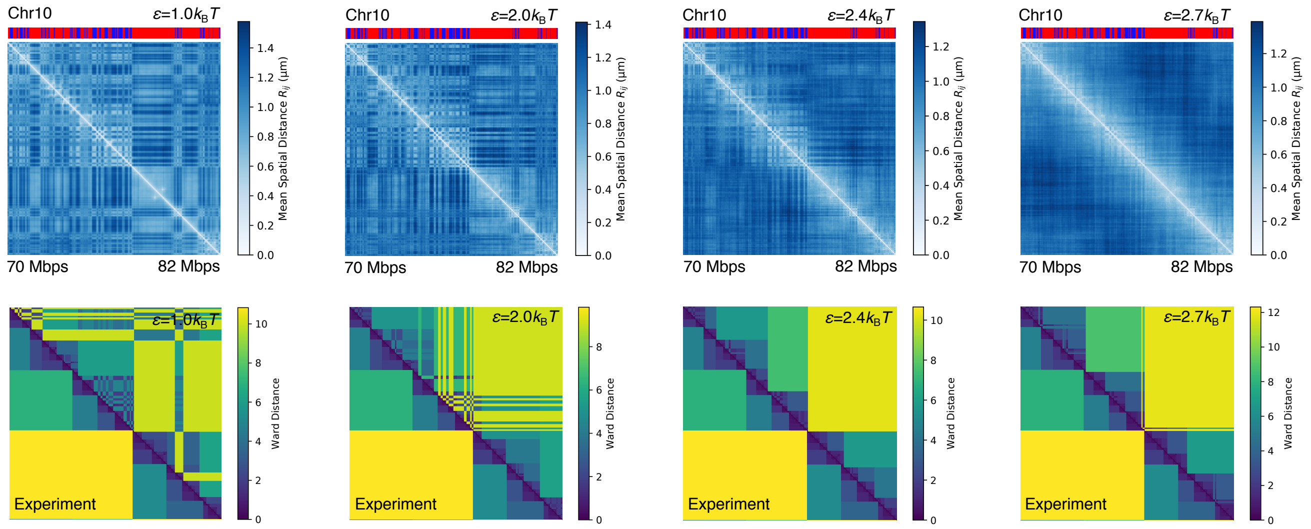

Supplementary Figure 16: Structural organization of Chromosome 10. **(Upper panel)** Distance maps of Chr10 for $\epsilon = (1.0, 2.0, 2.4, 2.7)k_{\mathrm{B}}T$. **(Lower panel)** Comparison between the experimental WLM (lower triangle) and the simulated Ward Linkage Matrix (WLMs) (upper triangle). Just as we found in Chr5, $\epsilon = 2.4k_{\mathrm{B}}T$ provides the best comparison with experiment, implying that the CCM is transferable.

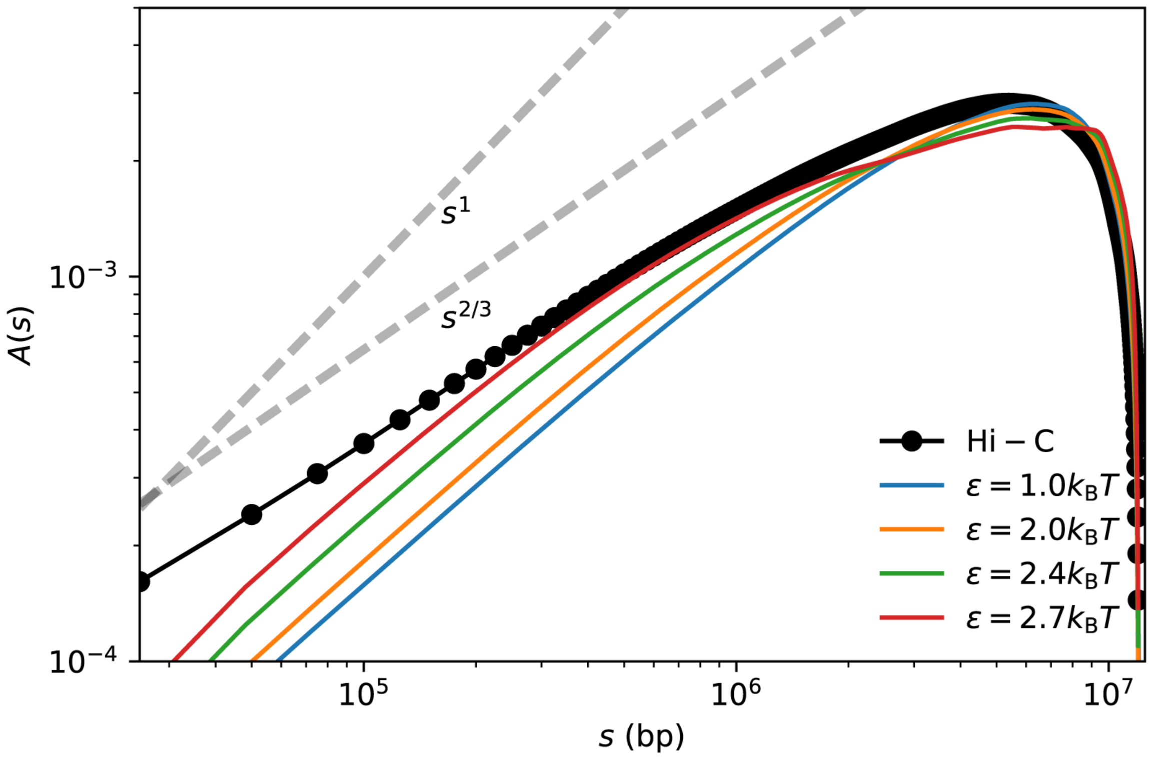

Supplementary Figure 17: Normalized surface contacts, $A(s)$, obtained using simulations and inferred from the Hi-C data; $A(s)$ is the normalized number of contacts between a segment of size s and the rest of the chromosome, given by $A(s) = (1/(N-s)\sum_{i<j} A_{ij})\sum_{i<j}(\sum_{l<i}\sum_{k=i}^{k=j} C_{kl} + \sum_{l>j}\sum_{k=i}^{k=j} C_{kl})\delta(s-(j-i))$. The grey lines give the expected scalings.

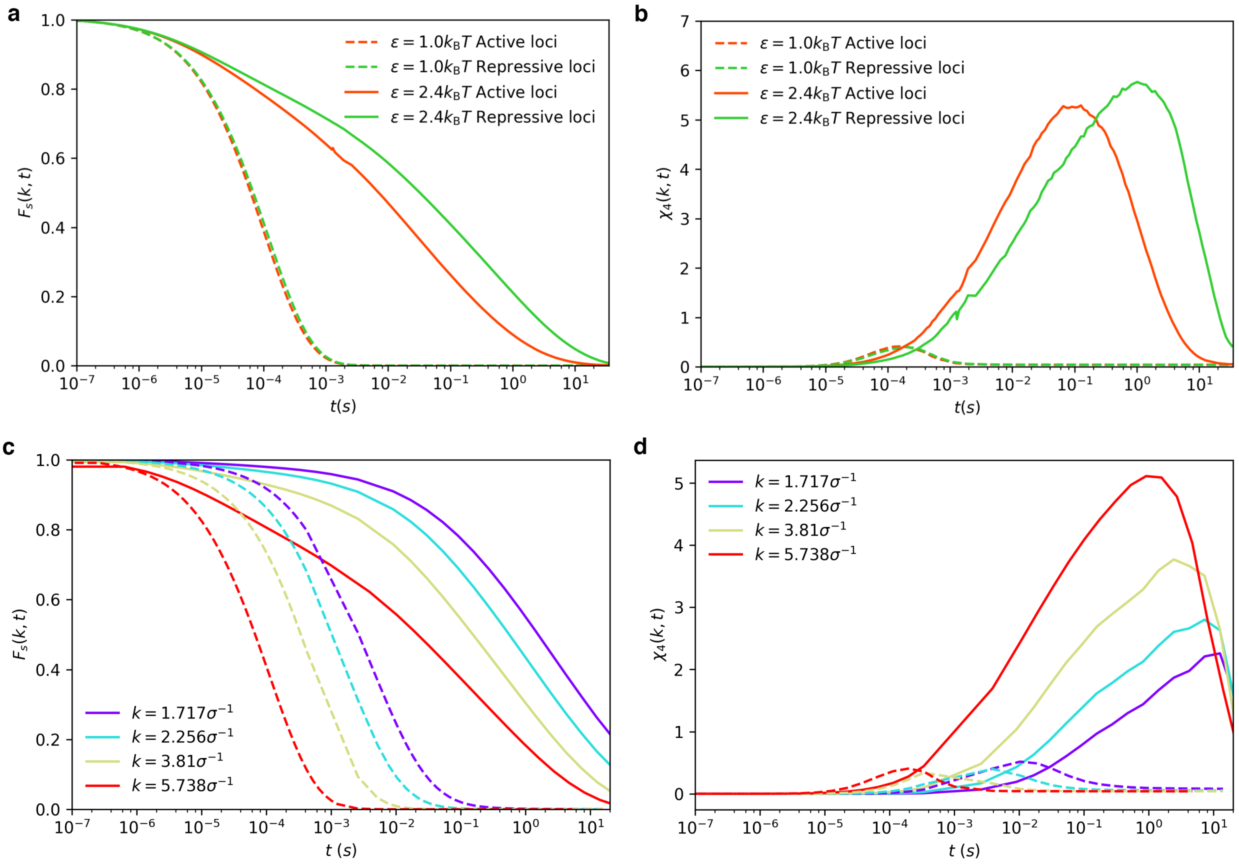

Supplementary Figure 18: Intermediate scattering function $F_s(k,t)$ (**a**) and $\chi_4(k,t)$ (**b**) for active (euchromatin) and repressive (heterochromatin) loci for Chr5. (**c**) and (**d**) show $F_s(k,t)$ and $\chi_4(k,t)$ for different values of k. In both **c** and **d**, dashed and solid curves are for $\epsilon = 1.0k_{\rm B}T$ and $\epsilon = 2.4k_{\rm B}T$, respectively. Curves with different colors are for $(k = 5.738, 3.281, 2.256, 1.717)\ \sigma^{-1}$ where $\sigma = 0.07\mu$m, corresponding to the first, second, third and fourth peak of the radial distribution function $g(r)$ (see Fig.2 in the main text). The relaxation times associated with the longest length scales is about an order of magnitude greater than the short length scale relaxation.

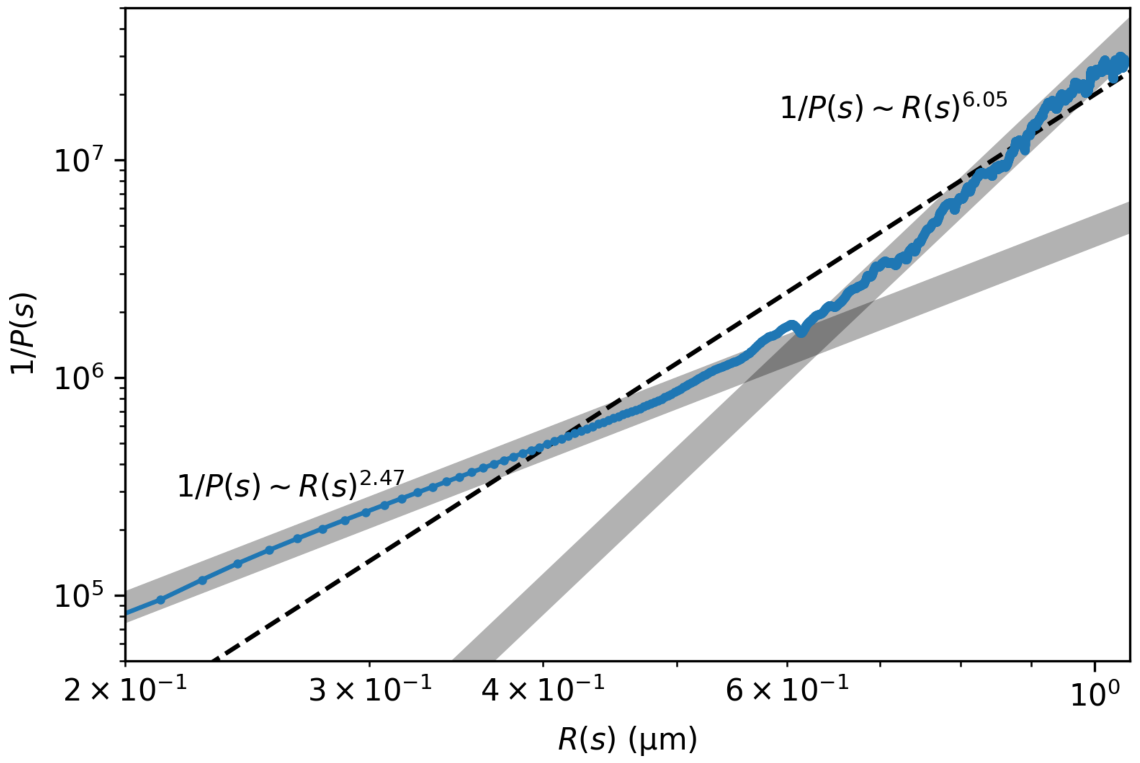

Supplementary Figure 19: The relation between $1/P(s)$ and $R(s)$ depends on s. $P(s) = \left\langle \sum_{i<j}^{N} \frac{\Theta(r_c - |\mathbf{r}_i - \mathbf{r}_j|)\delta(s-|i-j|)}{N-s} \right\rangle$, where $\Theta(x)$ is the Heaviside step function and r_c is the cutoff distance of contacts. $R(s) = \left\langle \sum_{i<j}^{N} \frac{(\mathbf{r}_i - \mathbf{r}_j)^2 \delta(s-|i-j|)}{N-s} \right\rangle^{1/2}$. $\langle \cdot \rangle$ is both ensemble and time average. Two thick gray lines are fit to the data, for $0.2\mu\text{m} \leq R(s) \leq 0.5\mu\text{m}$ and $0.7\mu\text{m} \leq R(s) \leq 1.0\mu\text{m}$, respectively. Dashed line has slope 4.1. The subtle relationship between $P(s)$ (Hi-C data) and $R(s)$ (FISH data) requires clarification (also see Ref. [20]).

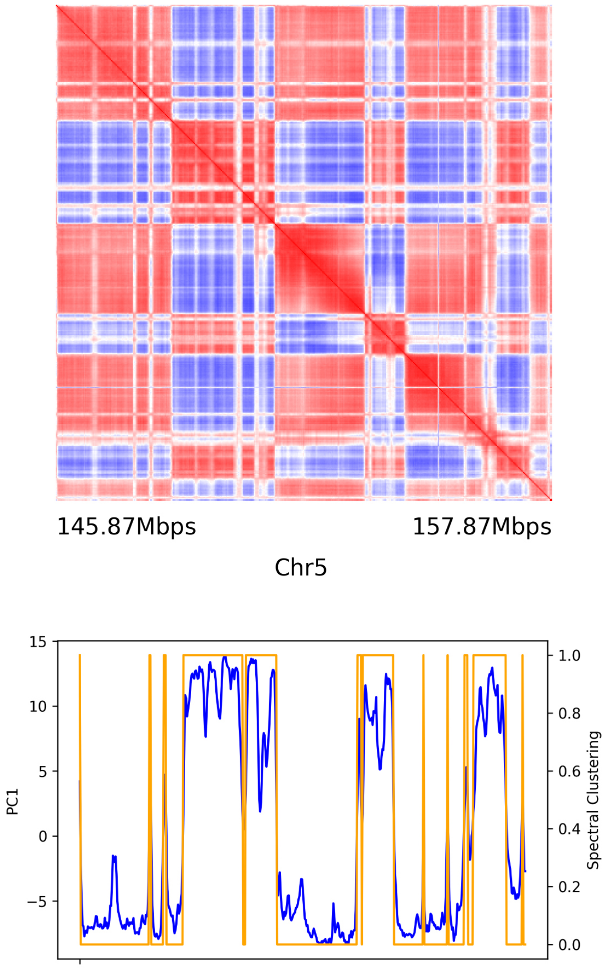

Supplementary Figure 20: Comparison between the Principle Components Analysis (PCA) and spectral co-clustering method. The upper panel shows the correlation matrix computed from experimental Hi-C data. The lower panel shows the results using the first principle component (blue) and the spectral co-clustering method (orange). It is clear that the results obtained from both methods are congruent.

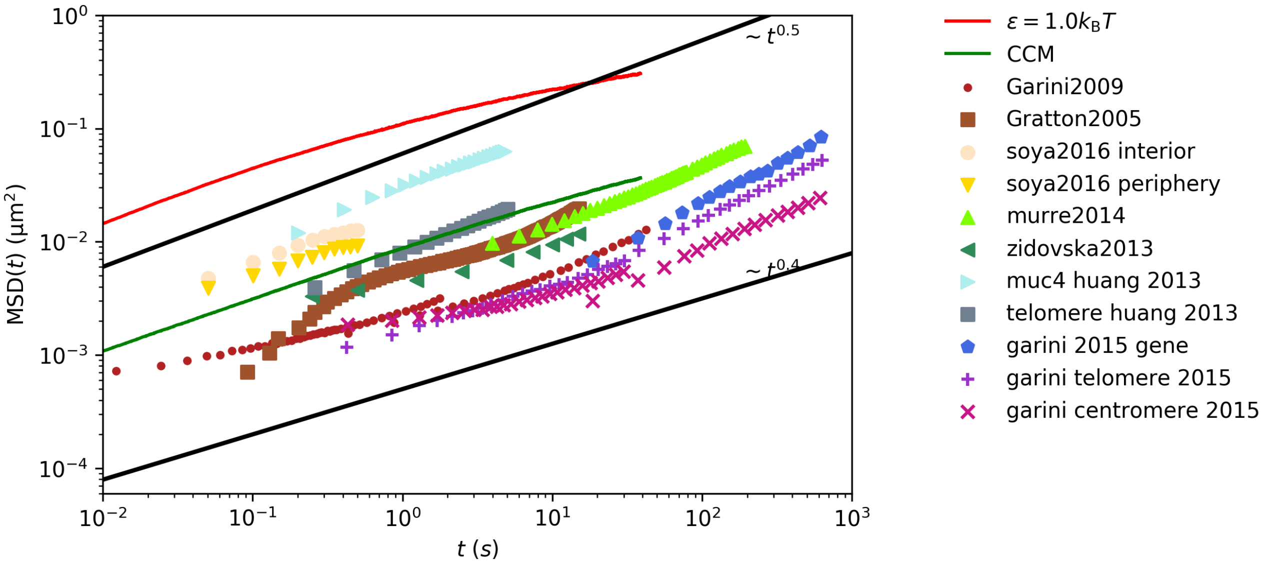

Supplementary Figure 21: MSD(t) experimental data collected from a number of works for human interphase cells. The simulation data for $\epsilon = 1.0k_BT$ and CCM is also plotted for comparison. The experimental data are taken from Bronstein et al., 2009 [21], Levi et al., 2005 [22], Shinkai et al., 2016 [23], Lucas et al., 2014 [24], Zidovska et al., 2013 [25], Chen et al., 2013 [26] and Bronshtein et al., 2015 [27]

43(B),396(B)	43(B),582(B)	143(B),396(B)	948(B),1110(B)
1407(B),1570(B)	1628(A),2120(B)	2355(B),2562(A)	2409(B),2562(A)
2622(A),2917(B)	3059(A),3106(B)	3307(A),3378(A)	3307(A),3630(B)
3307(A),3471(B)	4131(B),4175(A)	4131(B),4307(A)	4445(A),5012(B)
4445(A),4710(B)	5058(B),5548(B)	6318(B),6766(B)	6318(B),6408(B)
6318(B),6595(A)	6408(B),6595(A)	6647(B),6766(B)	7605(B),8907(A)
7917(B),8644(B)	7917(B),8743(A)	7917(B),8907(A)	8743(A),8907(A)
8921(B),9008(B)	9157(B),9396(A)	9481(A),9562(A)	9510(A),9562(A)

Supplementary Table 1: Loop anchor indices for Chromosome 5 (Chr 5) derived from the experimental data [1] for use in the CCM. Each pair of numbers represents single loop corresponding to the locations of the loop anchors along the backbone of the copolymer. The letter A (B) after each number indicates the type of the loop anchor. The number of loops in the simulation using the CCM is thirty-two. Fifteen out of thirty-two pairs have loop anchors formed from loci of the same type.

$K_{\rm S}/k_{\rm B}T\sigma^{-2}$	R_0/σ	$K_{\rm L}/k_{\rm B}T\sigma^{-2}$	$\epsilon_{AA}/k_{\rm B}T$	$\epsilon_{BB}/k_{\rm B}T$	$\epsilon_{AB}/k_{\rm B}T$	a/σ
30	1.5	300	1.0	1.0	0.82	1.13
30	1.5	300	2.0	2.0	1.64	1.13
30	1.5	300	2.4	2.4	1.96	1.13
30	1.5	300	2.7	2.7	2.21	1.13

Supplementary Table 2: Parameters values in the CCM for Chr5 and 10. Energy is in the unit of $k_{\rm B}T$ ($k_{\rm B}$ is the Boltzmann constant and T is the room temperature 300K), bead diameter σ is used as a measure of length. Without loss of generality, we choose $\epsilon_{AA} = \epsilon_{BB} = \epsilon$.

637(A),818(B)	637(A),960(A)	637(A),711(A)	831(B),960(A)
1924(A),2099(B)	1924(A),2199(A)	2146(B),2199(A)	2238(B),2474(B)
2620(B),2774(B)	2620(B),2890(B)	3096(B),3173(B)	3436(B),3828(A)
4186(B),4503(A)	4407(B),4503(A)	4674(A),4704(B)	4674(A),4750(B)
4704(B),4867(A)	4704(B),4750(B)	6922(B),7287(B)	9278(B),9356(B)
9435(A),9741(B)	9919(B),9985(A)	9940(A),9985(A)	

Supplementary Table 3: Loop anchor indices derived from the experimental data [1] for use in the CCM for Chr 10. Each pair of numbers represents single loop corresponding to the locations of the loop anchors along the backbone of the copolymer. The letter A (B) after each number indicates the type of the loop anchor.

Supplementary References

1. Rao, S. S. *et al.* A 3D Map of the Human Genome at Kilobase Resolution Reveals Principles of Chromatin Looping. *Cell* **159,** 1665–1680 (2014).
2. Jost, D., Carrivain, P., Cavalli, G. & Vaillant, C. Modeling epigenome folding: Formation and dynamics of topologically associated chromatin domains. *Nucleic Acids Res.* **42,** 9553–9561 (2014).
3. Michieletto, D., Orlandini, E. & Marenduzzo, D. Polymer model with Epigenetic Recoloring Reveals a Pathway for the *de novo* Establishment and 3D Organization of Chromatin Domains. *Phys. Rev. X* **6** (2016).
4. Rosenbloom, K. R. *et al.* ENCODE Data in the UCSC Genome Browser: year 5 update. *Nucleic Acids Res.* **41,** D56–D63 (2012).
5. Ernst, J. & Kellis, M. Discovery and characterization of chromatin states for systematic annotation of the human genome. *Nat. Biotechnol.* **28,** 817–825 (2010).
6. Ernst, J. *et al.* Mapping and analysis of chromatin state dynamics in nine human cell types. *Nature* **473,** 43–49 (2011).
7. Plimpton, S. Fast Parallel Algorithms for Short-Range Molecular Dynamics. *J. Comput. Phys.* **117,** 1–19 (1995).
8. *LAMMPS* http://lammps.sandia.gov. Accessed: 2017-08-01. 2017.
9. Honeycutt, J. D. & Thirumalai, D. The nature of folded states of globular proteins. *Biopolymers* **32,** 695–709 (1992).
10. Dixon, J. R. *et al.* Topological domains in mammalian genomes identified by analysis of chromatin interactions. *Nature* **485,** 376–380 (2012).
11. Wang, S. *et al.* Spatial organization of chromatin domains and compartments in single chromosomes. *Science* **353,** 598–602 (2016).
12. Dhillon, I. S. *Co-clustering documents and words using bipartite spectral graph partitioning* in *Proceedings of the seventh ACM SIGKDD international conference on Knowledge discovery and data mining* (2001), 269–274.
13. Vinh, N. X., Epps, J. & Bailey, J. Information theoretic measures for clusterings comparison: Variants, properties, normalization and correction for chance. *Journal of Machine Learning Research* **11,** 2837–2854 (2010).

14. Indyk, P. & Motwani, R. *Approximate nearest neighbors: towards removing the curse of dimensionality* in *Proceedings of the thirtieth annual ACM symposium on Theory of computing* (1998), 604–613.

15. Chung, M. K., Lee, H., Solo, V., Davidson, R. J. & Pollak, S. D. in *Connectomics in NeuroImaging* 161–170 (Springer International Publishing, 2017).

16. Müllner, D. Modern hierarchical, agglomerative clustering algorithms. Preprint at https://arxiv.org/abs/1109.2378.

17. Aronovitz, J. & Nelson, D. Universal features of polymer shapes. *J. Phys.* **47,** 1445–1456 (1986).

18. Dima, R. I. & Thirumalai, D. Asymmetry in the Shapes of Folded and Denatured States of Proteins. *J. Phys. Chem. B* **108,** 6564–6570 (2004).

19. Des Cloizeaux, J. Short range correlation between elements of a long polymer in a good solvent. *J. Phys.* **41,** 223–238 (1980).

20. Fudenberg, G. & Imakaev, M. FISH-ing for captured contacts: towards reconciling FISH and 3C. *Nat. Methods* **14,** 673–678 (2017).

21. Bronstein, I. *et al.* Transient Anomalous Diffusion of Telomeres in the Nucleus of Mammalian Cells. *Phys. Rev. Lett.* **103** (2009).

22. Levi, V., Ruan, Q., Plutz, M., Belmont, A. S. & Gratton, E. Chromatin Dynamics in Interphase Cells Revealed by Tracking in a Two-Photon Excitation Microscope. *Biophys. J.* **89,** 4275–4285 (2005).

23. Shinkai, S., Nozaki, T., Maeshima, K. & Togashi, Y. Dynamic Nucleosome Movement Provides Structural Information of Topological Chromatin Domains in Living Human Cells. *PLoS Comput Biol.* **12,** e1005136 (2016).

24. Lucas, J. S., Zhang, Y., Dudko, O. K. & Murre, C. 3D Trajectories Adopted by Coding and Regulatory DNA Elements: First-Passage Times for Genomic Interactions. *Cell* **158,** 339–352 (2014).

25. Zidovska, A., Weitz, D. A. & Mitchison, T. J. Micron-scale coherence in interphase chromatin dynamics. *Proc. Natl. Acad. Sci. U. S. A.* **110,** 15555–15560 (2013).

26. Chen, B. *et al.* Dynamic Imaging of Genomic Loci in Living Human Cells by an Optimized CRISPR/Cas System. *Cell* **155,** 1479–1491 (2013).

27. Bronshtein, I. *et al.* Loss of lamin A function increases chromatin dynamics in the nuclear interior. *Nat. Commun.* **6** (2015).
