# Supplementary material for: Interphase human chromosome exhibits out of equilibrium glassy dynamics
Source: Nat Commun. 2018 Aug 8;9:3161. doi: 10.1038/s41467-018-05606-6 (PMC6082855; doi:10.1038/s41467-018-05606-6)
Supplement: Supplementary file 2 — Description of Additional Supplementary Files [file 41467_2018_5606_MOESM2_ESM.pdf]

Description of Additional Supplementary Files

File Name: Supplementary Movie 1
Description: This movie shows the organization process of chromosome. The coloring of segments corresponds to genomic distance from one endpoint, ranging from red to green to blue.
